# Modified furosemide responsiveness index and biomarkers for AKI progression and prognosis: a prospective observational study

**DOI:** 10.1186/s13613-024-01387-y

**Published:** 2024-10-08

**Authors:** Ying Su, Wen-jun Liu, Yu-feng Zhao, Yi-jie Zhang, Yue Qiu, Zhi-hui Lu, Peng Wang, Shuang Lin, Guo-wei Tu, Zhe Luo

**Affiliations:** 1 Cardiac Intensive Care Center, Zhongshan Hospital, Fudan University, No. 180 Fenglin Road, Xuhui District, Shanghai, 200032 China; 2 Department of Urology, Zhongshan Hospital, Fudan University, Shanghai, China; 3 Shanghai Key Laboratory of Lung Inflammation and Injury, Shanghai, China; 4 Department of Critical Care Medicine, Zhongshan-Xuhui Hospital, Shanghai Xuhui Central Hospital, Fudan University, Shanghai, China

**Keywords:** Acute kidney injury, AKI progression, Renal biomarker, Modified furosemide responsiveness index, Furosemide stress test, Cystatin C, N-acetyl-β-D-glycosaminidase, Neutrophil gelatinase-associated lipocalin, Inflammation cytokines

## Abstract

**Background:**

Modified furosemide responsiveness index (mFRI) is a novel biomarker for assessing diuretic response and AKI progression in patients with early AKI. However, the comparative predictive performance of mFRI and novel renal biomarkers for adverse renal outcomes remains unclear. In a single-center prospective study, we aimed to evaluate the discriminatory abilities of mFRI and other novel renal biomarkers in predicting AKI progression and prognosis in patients with initial mild and moderate AKI (KDIGO stage 1 to 2).

**Results:**

Patients with initial mild and moderate AKI within 48 h following cardiac surgery were included in this study. The mFRI, renal biomarkers (including serum or urinary neutrophil gelatinase-associated lipocalin [sNGAL or uNGAL], serum cystatin C, urinary N-acetyl-beta-D-glycosaminidase [uNAG], urinary albumin-to-creatinine ratio) and cytokines (TNF, IL-1β, IL-2R, IL-6, IL-8, and IL-10) were measured at AKI diagnosis. The mFRI was calculated for each patient, which was defined as 2-hour urine output divided by furosemide dose and body weight. Of 1013 included patients, 154 (15.2%) experienced AKI progression, with 59 (5.8%) progressing to stage 3 and 33 (3.3%) meeting the composite outcome of hospital mortality or receipt of renal replacement therapy (RRT). The mFRI showed non-inferiority or potential superiority to renal biomarkers and cytokines in predicting AKI progression (area under the curve [AUC] 0.80, 95% confidence interval [CI] 0.77–0.82), progression to stage 3 (AUC 0.87, 95% CI 0.85–0.89), and composite outcome of death and receipt of RRT (AUC 0.85, 95% CI 0.82–0.87). Furthermore, the combination of a functional biomarker (mFRI) and a urinary injury biomarker (uNAG or uNGAL) resulted in a significant improvement in the prediction of adverse renal outcomes than either individual biomarker (all *P* < 0.05). Moreover, incorporating these panels into clinical model significantly enhanced its predictive capacity for adverse renal outcomes, as demonstrated by the C index, integrated discrimination improvement, and net reclassification improvement (all *P* < 0.05).

**Conclusions:**

As a rapid, cost-effective and easily accessible biomarker, mFRI, exhibited superior or comparable predictive capabilities for AKI progression and prognosis compared to renal biomarkers in cardiac surgical patients with mild to moderate AKI.

**Trial registration:**

Clinicaltrials.gov, NCT04962412. Registered July 15, 2021, https://clinicaltrials.gov/ct2/show/NCT04962412?cond=NCT04962412&draw=2&rank=1.

**Graphical abstract:**

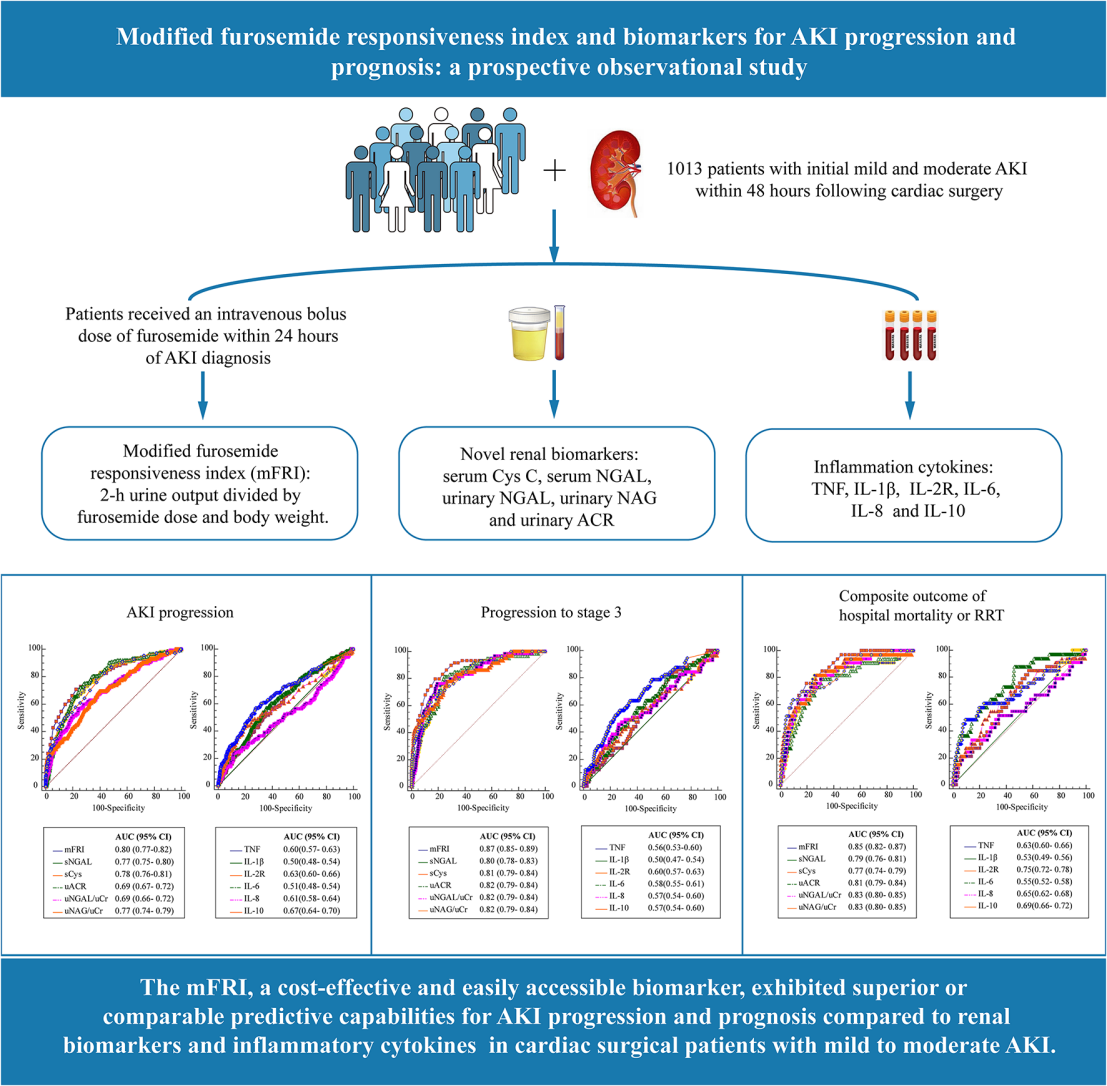

**Supplementary Information:**

The online version contains supplementary material available at 10.1186/s13613-024-01387-y.

## Highlights

The mFRI is a rapid, cost-effective and easily accessible biomarker.

The mFRI exhibited superior or comparable predictive capabilities for AKI progression and prognosis compared to renal biomarkers and cytokines in cardiac surgical patients with mild to moderate AKI.

Combining the functional biomarker mFRI with a urinary injury biomarker (uNAG or uNGAL) enhanced predictive accuracy for adverse renal outcomes.

**Supplementary Information:**

The online version contains supplementary material available at 10.1186/s13613-024-01387-y.

## Background

Acute kidney injury (AKI) is a common complication in patients undergoing cardiac surgery and is associated with a risk of chronic kidney disease (CKD), cardiovascular events, and long-term mortality [1, 2]. The risk of adverse events escalates with the progression of AKI stages [3]. Early identification of patients at high risk for AKI progression would facilitate targeted interventions to mitigate the risk of adverse renal outcomes [4]. Previous studies have shown that renal injury biomarkers like serum neutrophil gelatinase-associated lipocalin (sNGAL), urinary kidney injury molecule-1 (KIM-1), urinary matrix metalloproteinase-7, urinary insulin-like growth factor-binding protein 7 (IGFBP7) and tissue inhibitor of metalloproteinases-2 (TIMP-2) as well as inflammation biomarkers such as plasma IL-8 and urinary IL-18, have varying predictive abilities for AKI progression [5–10]. The availability of these novel biomarkers may be limited due to their expense or variable predictive performance. Recently, we proposed a new biomarker to quantify the diuretic response, referred to as modified furosemide responsiveness index (mFRI), by calculating the ratio of 2-hour urine output to nonstandardized furosemide dose and body weight [11]. The mFRI was inversely associated with risk of AKI progression in patients with early and moderate AKI in two independent cohort [11]. Sensitivity and specificity were 70.0% (95% confidence interval [CI] 58.7–79.7%) and 81.9% (95% CI 77.8–85.4%) at a cutoff value of 0.12 mL/(mg·kg)/2 h, respectively. The mFRI could serve as a cost-effective and readily available biomarker for identifying high-risk patients susceptible to AKI progression. To date, there exists a gap in the literature regarding the validation of mFRI in comparison with novel renal biomarkers for the prediction of AKI progression and prognosis. In present study, we aimed to evaluate the performance of mFRI alongside novel renal biomarkers, cytokines, and their combinations in predicting adverse renal outcomes, including AKI progression, AKI progression to stage 3, and a composite outcome of hospital mortality and the need for renal replacement therapy (RRT).

## Methods

### Study population

This single-center prospective study included consecutive patients who underwent cardiac surgery at the cardiac intensive care unit of Zhongshan Hospital, Fudan University, China, between February 1, 2022, and October 31, 2022. The study protocol was approved by the Ethics Committee of Zhongshan Hospital, Fudan University (B2021-390R) and registered at ClinicalTrials.gov (NCT04962412). The study was conducted in accordance with the Declaration of Helsinki, and written informed consent was obtained from the legal representatives of the patients.

Inclusion criteria comprised adult patients aged 18 years or older who developed mild to moderate AKI (stage 1 or 2) within 48 h of cardiac surgery and received an intravenous bolus dose of furosemide within 24 h of AKI identification. Patients with pre-existing chronic kidney disease (preoperative estimated glomerular filtration rate [eGFR] < 30 mL/min/1.73 m^2^) or previous RRT before cardiac surgery, a history of kidney transplant or other kidney diseases, known pregnancy, multiple operation during the hospital stay, absence of furosemide administration or a delay exceeding 24 h from AKI criteria to furosemide administration, continuous furosemide infusion prior to or within 2 hours after the initial bolus dose, previous administration of bolus furosemide within 6 h of the first dose, repeated use of furosemide within 2 hours after the first dose, unavailable serum or urine samples, missing data on urine output after furosemide administration, initial AKI stage 3 within 48 h of cardiac surgery or being in a moribund state (with an anticipated likelihood of death within 24 h) were excluded from the study.

### Data collection

The following data were collected: demographic characteristics, comorbidities, baseline renal function, vital signs at AKI diagnosis, as well as serum daily creatinine, RRT, and death. The available data on furosemide dose, administration time, and hourly urine output were also extracted.

AKI was defined based on the Kidney Disease Improving Global Outcomes (KDIGO) criteria, which include both serum creatinine (SCr) and urine output criteria. The baseline serum creatinine was determined as the lowest available value within 3 months preceding cardiac surgery. If preoperative SCr level was not available, the first SCr measured at hospital admission was used as the baseline SCr. The preoperative eGFR was calculated using the modification of diet in renal disease (MDRD) equation.

### Biomarker arrays

The mFRI was determined by dividing the total urine output in a 2-hour period by the dose of intravenous bolus furosemide administered within 24 h of AKI diagnosis and the patient’s body weight [mL/(mg·kg)/2 h] [11]. The blood and urine samples were obtained within 6 h after AKI diagnosis, centrifuged, flash-frozen, stored at -80 °C, and thawed immediately prior to analysis. All biomarker measurements were conducted in the central laboratory at Zhongshan hospital, with laboratory personnel blinded to patient outcomes. The serum or urinary NGAL (sNGAL or uNGAL), serum cystatin C (sCys C), urinary N-acetyl-beta-D-glycosaminidase (uNAG), urinary albumin, urinary and serum creatinine were measured using the LABOSPECT 008AS platform (Hitachi High-Tech Co., Tokyo, Japan) according to the manufacturer’s instructions. The coefficients of inter-assay and intra-assay variation for sNGAL, uNGAL, sCysC and uNAG ranged 3–6% and 4–9%, respectively. All urinary biomarkers were normalized for urinary creatinine. Circulating cytokines including tumor necrosis factor (TNF), interleukin-1β (IL-1β), interleukin-2 receptor (IL-2R), interleukin-6 (IL-6), interleukin-8 (IL-8) and interleukin-10 (IL-10) were measured using the Immulite 1000 immunoassay system (Siemens, Munich, Germany). The inter-assay and intra-assay coefficients of variation for cytokines were both < 10%.

### Outcomes

The primary outcome was AKI progression, defined as worsening of AKI stage within 1 week (progressing from stage 1 to either stage 2 or stage 3 or from stage 2 to stage 3). The other outcomes were monitored: progression to stage 3, composite outcome of hospital mortality or receipt of RRT, duration of mechanical ventilation, length of ICU and hospital stay.

### Statistical analysis

Patient characteristics were reported as median (interquartile range [IQR]) or mean (standard deviation) for continuous variables, and as frequencies and proportions for categorical variables. Continuous variables were compared using Student’s t-test or Mann-Whitney U test, while categorical variables were analyzed using chi-square test or Fisher’s exact test, as appropriate. The Spearman correlation coefficient was used to assess the correlation between biomarkers.

The predictive ability of mFRI and biomarkers for AKI progression was assessed by calculating the area under the curve (AUC). The robustness of mFRI’s predictive value was validated by evaluating AKI progression to stage 3 and composite outcome of hospital mortality or receipt of RRT. AUC comparisons between groups were conducted using the DeLong method. Logistic regression models were employed to evaluate the discriminatory ability of panels of mFRI and other renal biomarkers in predicting adverse renal outcomes compared with the mFRI alone. The panels with highest AUC were chosen for subsequent analysis. Sensitivity, specificity, positive likelihood ratio, negative likelihood ratio, positive predictive value and negative predictive value of the biomarkers were calculated, and optimal cutoff values determined using Youden’s index. Besides, the logistic regression models were also used to evaluate associations between biomarkers and adverse renal outcomes, such as AKI progression, progression to stage 3, and composite outcomes. We adjusted for confounders using two models. Model 1 was unadjusted. Model 2 was adjusted for age, gender, Body Mass Index (BMI), diabetes mellitus, hypertension, coronary artery disease (CAD), cerebrovascular disease, preoperative diuretic exposure, baseline eGFR, surgical type, cardiopulmonary bypass used, central venous pressure (CVP), AKI stage at enrollment and sequential organ failure assessment (SOFA) score [11]. The performance of mFRI and combined biomarker panels was compared to a reference clinical model using C-index, integrated discrimination improvement (IDI), and net reclassification improvement (NRI) indices. Statistical analyses were conducted using SPSS 24.0 (IBM, Armonk, NY, USA) and R software (R Foundation for Statistical Computing), with a significance level set at *P* < 0.05.

## Results

### Patient characteristics

Between February 1, 2022, and October 31, 2022, a total of 3,566 consecutive adult cardiac patients underwent prospective screening for this study. Among them, 2,553 patients were excluded for various reasons, as outlined in Fig. S1 in Supplementary Material 1. Ultimately, 1,013 patients who met mild to moderate AKI criteria after cardiac surgery were included in the analysis. The characteristics of the patients are presented in Table 1.

**Table 1:** Demographic and clinical characteristics of patients

	Overall (*n* = 1013)	AKI without progression (*n* = 859)	AKI progression (*n* = 154)	*P* value
Age, years	62[53,69]	63[53,69]	61[51,70]	0.64
Male sex, n (%)	785 (77.5%)	670 (78.0%)	115 (74.7%)	0.36
Height, cm	168[161,172]	168[161,172]	168[162,172]	0.93
Weight, kg	68.5[60,76]	68.5[60,76]	68.5[59.75,80]	0.60
BMI, kg/m²	24.49[22.31,26.75]	24.46[22.28,26.67]	24.58[22.32,27.39]	0.42
**Comorbidities**				
Diabetes mellitus, n (%)	166 (16.4%)	146 (17.0%)	20 (13.0%)	0.22
Hypertension, n (%)	546 (53.9%)	460(53.6%)	86(55.8%)	0.60
CAD, n (%)	208 (20.5%)	175 (20.4%)	33 (21.4%)	0.77
COPD, n (%)	1 (0.1%)	1 (0.1%)	0 (0.0%)	1.00
Cerebrovascular disease, n (%)	62 (6.1%)	49 (5.7%)	13 (8.4%)	0.19
Preoperative diuretic exposure, n (%)	615 (60.7%)	526 (61.2%)	89 (57.8%)	0.42
Baseline blood urea nitrogen, mmol/L	6.8[5.7,8.3]	6.8[5.7,8.2]	6.8[5.8,9.13]	0.08
Baseline creatinine, µmol/L	85[73,100]	84.5[73,98]	91[76,108.25]	< 0.01
Baseline eGFR, ml/min/1.73m^2^	80[65,96]	81[66.25,97]	74[60.75,88.25]	< 0.01
**Type of surgery**,** n (%)**				< 0.001
CABG only	124 (12.2%)	108 (12.6%)	16 (10.4%)	
Valve only	561 (55.4%)	498 (58.0%)	63 (40.9%)	
CABG and valve	70 (6.9%)	53 (6.2%)	17 (11.0%)	
Aortic surgery	197 (19.4%)	152(17.7%)	45 (29.2%)	
Other cardiac surgery	59 (5.8%)	46 (5.4%)	13 (8.4%)	
**Procedural characteristics**				-
Cardiopulmonary bypass used, n (%)	894 (88.3%)	752 (87.5%)	142 (92.2%)	0.10
Cardiopulmonary bypass time, min	125.5[98,162]	120[95,150]	169.5[131.75,209.25]	< 0.01
Cross-clamp time, min	73[55,96]	71[53,93]	87[68,111.5]	< 0.01
**Clinical Characteristics at enrollment**				
AKI stage at enrollment, n (%)				0.31
Stage 1	893 (88.2%)	761 (88.6%)	132 (85.7%)	
Stage 2	120 (11.8%)	98 (11.4%)	22 (14.3%)	
**Vital signs**				
Heart rate, beats/minute	83[76,93]	83[75,93]	83[79,96]	0.15
MAP, mm Hg	77.67[71.33,85]	78.67[72,85.33]	73.33[67.33,81.17]	< 0.01
CVP, mm Hg	11[10,13]	11[10,12]	12[11,14]	< 0.01
Invasive mechanical ventilation, n (%)	409 (40.4%)	296 (34.5%)	113 (73.4%)	< 0.001
Need for vasopressor support, n (%)	657 (64.9%)	532 (61.9%)	125 (81.2%)	< 0.001
SOFA score, points	4[2,6]	4[2,6]	7[5,9]	< 0.01
Furosemide dose, mg	20[20,20]	20[20,20]	20[20,40]	< 0.01
2 h urine output, mL	285[180,420]	300[200,450]	150[88.75,250]	< 0.01
mFRI, mL/(mg·kg)/2 h	0.20 [0.12,0.32]	0.22[0.14,0.34]	0.08[0.04,0.16]	< 0.01
**Outcome**				
AKI Progression, n (%)	154(15.2%)	0 (0.0%)	154(100%)	< 0.001
Progression to stage 3, n (%)	59 (5.8%)	0 (0.0%)	59 (38.3%)	< 0.001
RRT, n (%)	16 (1.6%)	0 (0.0%)	16 (10.4%)	< 0.001
Duration of invasive mechanical ventilation, hours	19[15,36]	18[15,22]	46[19.75,108.5]	< 0.001
Median length of ICU stay, days	2.2[1,4.7]	1.9[1,3.8]	6[3.8,11.05]	< 0.001
Median length of hospital stay, days	11.5[8.9,14.5]	10.8[8.8,13.8]	14.2[10.98,20.18]	< 0.001
Hospital mortality, n (%)	26 (2.6%)	7 (0.8%)	19 (12.3%)	< 0.001
Composite outcome of RRT or death, n (%)	33 (3.3%)	7 (0.8%)	26 (16.9%)	< 0.001

Data are presented as median [inter-quartile range] or n (%). AKI, acute kidney injury, BMI, body mass index, CABG, coronary artery bypass grafting, CAD, coronary artery disease, COPD, chronic obstructive pulmonary disease, CVP, central venous pressure, eGFR, estimated glomerular filtration rate, MAP, mean arterial pressure, HR, heart rate, ICU, intensive care unit, mFRI, modified furosemide responsiveness index, SOFA score, sequential organ failure assessment score, RRT, renal replacement therapy

Patients with progressive AKI exhibited certain distinct characteristics compared to those without progression (Table 1). Specifically, they had lower baseline eGFR, higher rates of redo cardiac surgery, longer durations of cardiopulmonary bypass and cross-clamp time. Moreover, these patients demonstrated evidence of more severe conditions at enrollment, as indicated by higher SOFA scores, elevated CVP, increased rates of invasive mechanical ventilation, and a need for vasopressor support.

### Prediction of AKI progression

**Fig. 1:**
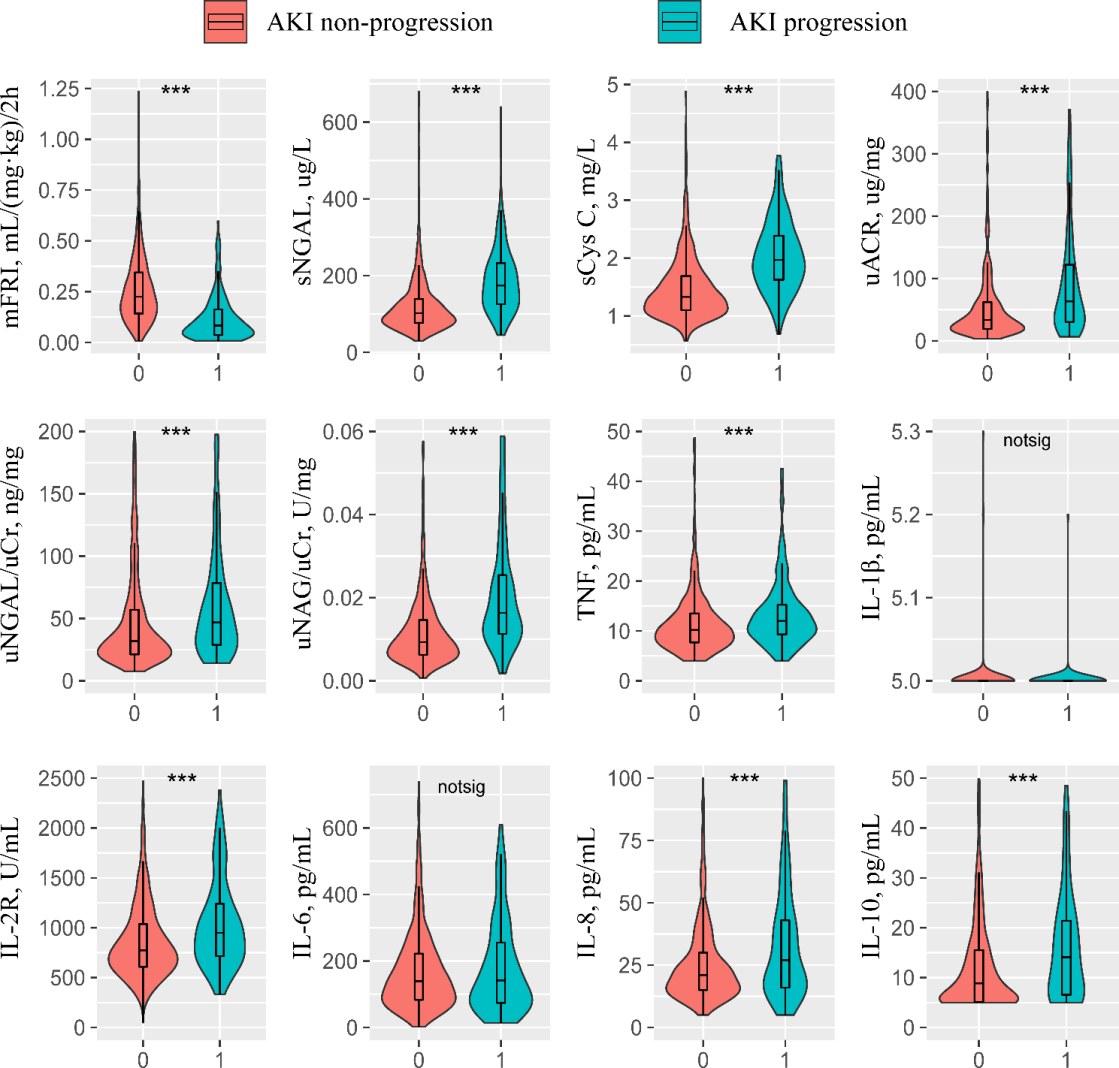
Comparisons of biomarkers between patients with AKI progression and those without AKI progression. *** indicates *P* < 0.001, notsig indicates not statistically significant. AKI, acute kidney injury, IL-1β, interleukin-1β, IL-2R, interleukin-2 receptor, IL-6, interleukin-6, IL-8, interleukin-8, IL-10, interleukin-10, mFRI, modified furosemide responsiveness index, sCysC, serum cystatin C, sNGAL, serum neutrophil gelatinase-associated lipocalin, TNF, tumor necrosis factor, uACR, urinary albumin/creatinine ratio, uCr, urinary creatinine, uNAG, urinary N-acetyl-β-D-glycosaminidase, uNGAL, urinary neutrophil gelatinase-associated lipocalin

Within a period of 7 days, 154 patients (15.2%) experienced progression to a higher severity of AKI. Patients who experienced AKI progression exhibited significantly lower values of the mFRI (*P* < 0.01), while significantly higher levels of five renal biomarkers, including sNGAL, sCys C, uACR, uNGAL/uCr, and uNAG/uCr, were observed in these patients compared to those who did not worsen (Fig. 1 and Table S1 in Supplementary Material 2, all *P* < 0.01). In patients with AKI progression, the levels of TNF, IL-2R, IL-8, and IL-10 were significantly higher compared to those without AKI progression (all *P* < 0.01). Conversely, there were no significant differences in the levels of IL-1β and IL-6 between the two groups. The mFRI was inversely correlated with other renal biomarkers including sNGAL, sCys C, uACR, uNGAL/uCr and uNAG/uCr (Supplementary Material 3: Fig. S2).

**Fig. 2:**
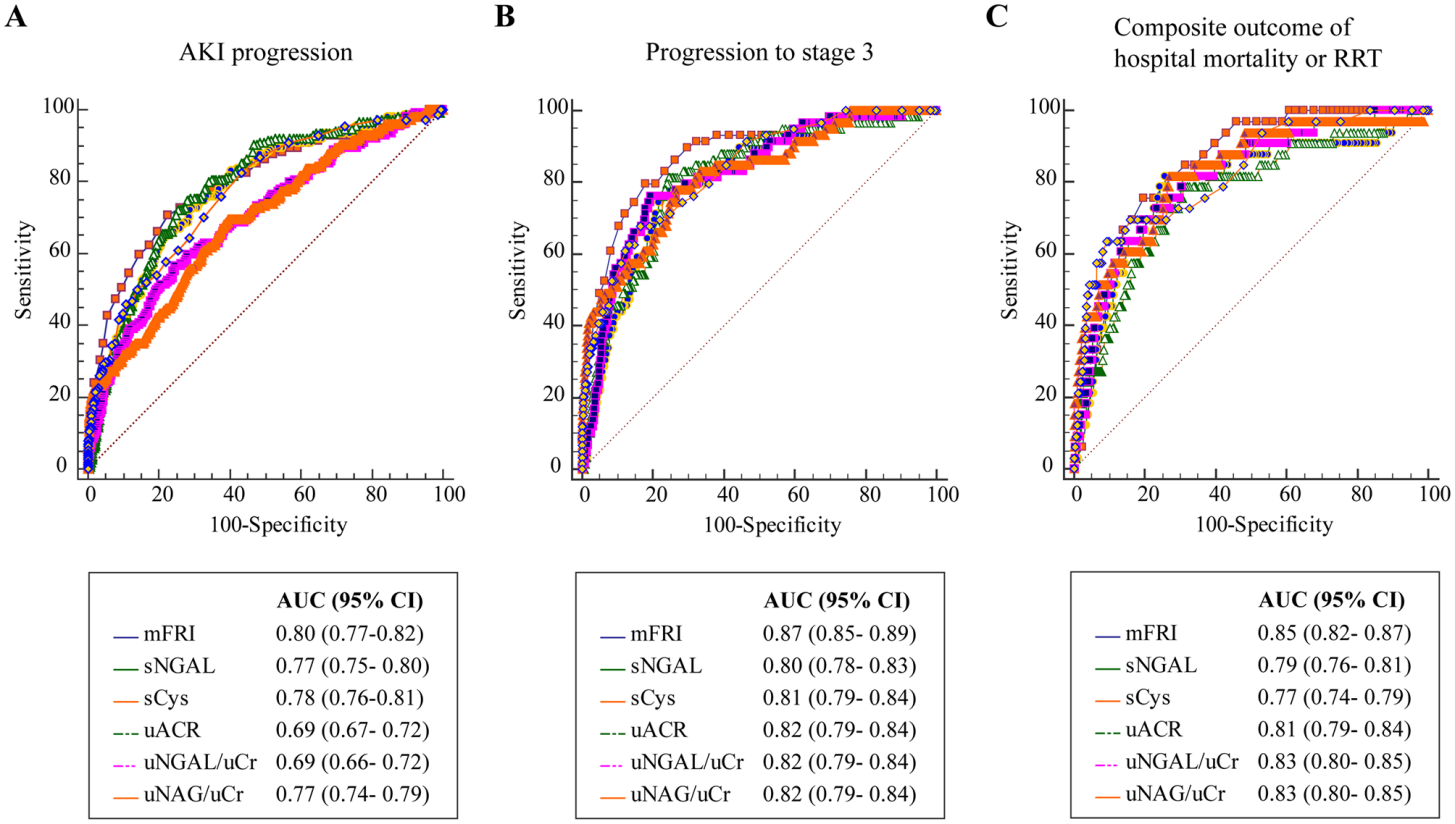
Predictive performance of biomarkers for AKI progression (**A**), progression to stage 3 (**B**) and composite outcome (**C**). AKI, acute kidney injury, AUC, area under the curve, CI, confidence interval, mFRI, modified furosemide responsiveness index, sCysC, serum cystatin C, sNGAL, serum neutrophil gelatinase-associated lipocalin, uACR, urinary albumin/creatinine ratio, uCr, urinary creatinine, uNAG, urinary N-acetyl-β-D-glycosaminidase, uNGAL, urinary neutrophil gelatinase-associated lipocalin, RRT, renal replacement therapy

The AUCs of mFRI, renal biomarkers and cytokines were calculated to evaluate the predictive performance for AKI progression (Fig. 2; Table 2). The mFRI demonstrated excellent performance in predicting AKI progression, with an AUC of 0.80 (95% CI 0.77–0.82, *P* < 0.001). And the renal biomarkers including sNGAL (AUC 0.77, 95% CI 0.75–0.80), sCys C (AUC 0.78, 95% CI 0.76–0.81), uACR (AUC 0.69, 95% CI 0.67–0.72), uNGAL/uCr (AUC 0.69, 95% CI 0.66–0.72), and uNAG/uCr (AUC 0.77, 95% CI 0.74–0.79) also showed significant predictive ability for AKI progression. Regarding cytokines, only TNF, IL-2R, IL-8 and IL-10 demonstrated low predictive potential for AKI progression (AUC range, 0.60–0.67). When comparing the AUCs head-to-head, the AUC of mFRI was significantly better than the other biomarkers, except for sNGAL, sCys C, and uNAG/uCr (Table 2). The optimal cutoff value of mFRI was 0.13 mL/(mg·kg)/2 h for predicting AKI progression, with sensitivity of 70.78% (95% CI 62.9–77.8) and specificity of 77.42% (95% CI 74.5–80.2).

**Table 2:** Predictive performance of biomarkers for AKI progression

Biomarkers	AUC ± SEM	95% CI	*P* Value	*P* Value Compared With mFRI	Cutoff	Sensitivity (95%CI)	Specificity (95%CI)	LR+ (95%CI)	LR- (95%CI)	PPV (95%CI)	NPV (95%CI)
Tubular function biomarker											
mFRI, mL/(mg·kg)/2 h	0.80 ± 0.02	0.77–0.82	< 0.01	-	0.13	70.78(62.9–77.8)	77.42(74.5–80.2)	3.13(2.7–3.7)	0.38(0.3–0.5)	36(30.6–41.7)	93.7(91.6–95.3)
**Traditional biomarker**											
uACR, ug/mg	0.69 ± 0.02	0.67–0.72	< 0.01	< 0.001	66.5	56.49(48.3–64.5)	75.2(72.2–78.1)	2.28(1.9–2.7)	0.58(0.5–0.7)	29.1(24.0–34.6)	90.6(88.2–92.6)
**GFR biomarker**											
sCys C, mg/L	0.78 ± 0.02	0.76–0.81	< 0.01	0.64	1.67	72.08(64.3–79.0)	74.5(71.4–77.4)	2.83(2.4–3.3)	0.37(0.3–0.5)	33.7(28.6–39.1)	93.7(91.6–95.4)
**Kidney injury biomarkers**											
sNGAL, ug/L	0.77 ± 0.02	0.75–0.80	< 0.01	0.34	130	72.73(65.0–79.6)	70.37(67.2–73.4)	2.45(2.1–2.8)	0.39(0.3–0.5)	30.7(26.0–35.7)	93.5(91.3–95.3)
uNGAL/uCr, ng/mg	0.69 ± 0.02	0.66–0.72	< 0.01	< 0.001	41.2	69.48(61.6–76.6)	60.49(57.1–63.8)	1.76(1.5–2.0)	0.5(0.4–0.6)	24.1(20.2–28.4)	91.7(89.1–93.8)
uNAG/uCr, U/mg	0.77 ± 0.02	0.74–0.79	< 0.01	0.33	0.01	82.47(75.5–88.1)	56.56(53.2–59.9)	1.9(1.7–2.1)	0.31(0.2–0.4)	25.5(21.7–29.6)	94.7(92.4–96.5)
**Circulating inflammatory biomarkers**											
TNF, pg/mL	0.6 ± 0.02	0.57–0.63	< 0.01	< 0.001	9.3	74.83(67.1–81.5)	41.98(38.6–45.4)	1.29(1.2–1.4)	0.6(0.5–0.8)	18.7(15.6–22.0)	90.4(87.0–93.1)
IL-1β, pg/mL	0.50 ± 0.03	0.48–0.54	0.77	< 0.001	5.0	80.13(72.9–86.2)	21.58(18.9–24.5)	1.02(0.9–1.1)	0.92(0.7–1.3)	15.4(12.9–18.1)	85.9(80.5–90.3)
IL-2R, U/mL	0.63 ± 0.02	0.60–0.66	< 0.01	< 0.001	865	60.26(52.0–68.1)	61.08(57.7–64.4)	1.55(1.3–1.8)	0.65(0.5–0.8)	21.6(17.8–25.9)	89.6(86.8–92.0)
IL-6, pg/mL	0.51 ± 0.03	0.48–0.54	0.77	< 0.001	308	23.84(17.3–31.4)	88.21(85.8–90.3)	2.02(1.4–2.8)	0.86(0.8–0.9)	26.5(19.3–34.7)	86.7(84.2–88.9)
IL-8, pg/mL	0.61 ± 0.03	0.58–0.64	< 0.01	< 0.001	34	43.71(35.7–52.0)	78.89(76.0–81.6)	2.07(1.7–2.6)	0.71(0.6–0.8)	26.9(21.5–33.0)	88.7(86.2–90.9)
IL-10, pg/mL	0.67 ± 0.03	0.64–0.70	< 0.01	< 0.001	15.7	56.95(48.7–65.0)	72.17(69.0–75.2)	2.05(1.7–2.4)	0.6(0.5–0.7)	26.7(22.0–31.9)	90.4(87.9–92.5)

AKI, acute kidney injury, AUC, area under the curve, CI, confidence interval, GFR, glomerular filtration rate, IL-1β, interleukin-1β, IL-2R, interleukin-2 receptor, IL-6, interleukin-6, IL-8, interleukin-8, IL-10, interleukin-10, mFRI, modified furosemide responsiveness index, SEM, standard error of mean, sCysC, serum cystatin C, sNGAL, serum neutrophil gelatinase-associated lipocalin, TNF, tumor necrosis factor, uACR, urinary albumin/creatinine ratio, uCr, urinary creatinine, uNAG, urinary N-acetyl-β-D-glucosaminidase, uNGAL, urinary neutrophil gelatinase-associated lipocalin, LR+, Positive likelihood ratio, LR-, negative likelihood ratio, PPV, Positive predictive value, NPV, Negative predictive value

To enhance the predictive performance of biomarkers for AKI progression, several panels incorporating mFRI and renal biomarkers (mFRI plus sNGAL, mFRI plus sCys C, mFRI plus uACR, mFRI plus uNGAL/uCr, and mFRI plus uNAG/uCr) were developed. The addition of other renal biomarkers significantly improved the AUC of mFRI (Table 3 and Fig. S3 in Supplementary Material 4). Among these panels, the combination of mFRI and uNAG/uCr exhibited the highest AUC values (AUC 0.83, 95% CI 0.80–0.85) for predicting AKI progression.

**Table 3:** Predictive performance of combined mFRI and renal biomarkers for AKI progression

Biomarkers	AUC ± SEM	95%CI	*P* Value	*P* Value Compared With mFRI	Cutoff	Sensitivity (95%CI)	Specificity (95%CI)	LR+ (95%CI)	LR- (95%CI)	PPV (95%CI)	NPV (95%CI)
Tubular function biomarker											
mFRI	0.80 ± 0.02	0.77–0.82	< 0.01	-	0.13	70.78(62.9–77.8)	77.42(74.5–80.2)	3.13(2.7–3.7)	0.38(0.3–0.5)	36(30.6–41.7)	93.7(91.6–95.3)
**Biomarker combination**											
mFRI + uACR	0.80 ± 0.02	0.78–0.83	< 0.01	0.02	0.20	72.08 (64.3–79.0)	76.84 (73.9–79.6)	3.11 (2.7–3.6)	0.36 (0.3–0.5)	35.9 (30.6–41.5)	93.9 (91.8–95.5)
mFRI + sCys C	0.82 ± 0.02	0.79–0.84	< 0.01	0.006	0.13	83.77 (77.0–89.2)	68.89 (65.7–72.0)	2.69 (2.4–3.0)	0.24 (0.2–0.3)	32.7 (28.1–37.5)	95.9 (94.0–97.3)
mFRI + sNGAL	0.82 ± 0.02	0.79–0.84	< 0.01	0.002	0.19	73.38 (65.7–80.2)	79.27 (76.4–81.9)	3.54 (3.0–4.2)	0.34 (0.3–0.4)	39 (33.3–44.8)	94.3 (92.3–95.9)
mFRI + uNGAL/uCr	0.82 ± 0.02	0.79–0.84	< 0.01	0.008	0.20	70.13 (62.2–77.2)	81.48 (78.7–84.0)	3.79 (3.2–4.5)	0.37 (0.3–0.5)	40.6 (34.6–46.8)	93.8 (91.8–95.4)
mFRI + uNAG/uCr	0.83 ± 0.02	0.80–0.85	< 0.01	0.001	0.18	70.78 (62.9–77.8)	81.5 (78.7–84.0)	3.83 (3.2–4.6)	0.36 (0.3–0.5)	40.8 (34.9–47.0)	93.9 (92.0–95.5)

AKI, acute kidney injury, AUC, area under the curve, CI, confidence interval, mFRI, modified furosemide responsiveness index, SEM, standard error of mean, sCysC, serum cystatin C, sNGAL, serum neutrophil gelatinase-associated lipocalin, uACR, urinary albumin/creatinine ratio, uCr, urinary creatinine, uNAG, urinary N-acetyl-β-D-glucosaminidase, uNGAL, urinary neutrophil gelatinase-associated lipocalin, LR+, Positive likelihood ratio, LR-, negative likelihood ratio, PPV, Positive predictive value, NPV, Negative predictive value

### Prediction of AKI progression to stage 3

Fifty-nine patients (5.8% of the total cohort) progressed to stage 3 within 7 days. These patients showed significantly lower mFRI values and significantly higher levels of sNGAL, sCys C, uACR, uNGAL/uCr and uNAG/uCr compared to those who did not worsen (Fig. S4 in Supplementary Material 5 and Table S1 in Supplementary Material 2, all *P* < 0.01). In terms of cytokines, patients with progression to stage 3 exhibited significantly higher levels of IL-2R, IL-6 and IL-10 (compared to those without progression to stage 3) (all *P* < 0.05). However, the levels of TNF, IL-1β and IL-8 were comparable between two groups.

The mFRI significantly outperformed sNGAL, sCys C and cytokines for predicting progression to stage 3 (Fig. 2 and Table S2 in Supplementary Material 2, all *P* < 0.05). Although the mFRI exhibited higher AUC (AUC 0.87, 95% CI 0.85–0.89) in predicting progression to stage 3 than uACR (AUC 0.82, 95% CI 0.79–0.84), uNGAL/uCr (AUC 0.82, 95% CI 0.79–0.84) and uNAG/uCr (AUC 0.84, 95% CI 0.81–0.85), the differences were not statistically significant. The optimal cutoff value of mFRI was 0.10 mL/(mg·kg)/2 h for predicting progression to stage 3, with sensitivity of 79.66% (95% CI 67.2–89.0) and specificity of 81.97% (95% CI 79.4–84.4).

We also examined the predictive value of combining biomarkers for AKI progression to stage 3. Among the evaluated panels, the combination of mFRI and uNGAL/uCr demonstrated the highest AUC values (AUC 0.91, 95% CI 0.89–0.92). The panels of mFRI plus uNGAL/uCr and mFRI plus uNAG/uCr significantly improved the AUC of mFRI in predicting progression to stage 3 (Fig. S3 in Supplementary Material 4 and Table S3 in Supplementary Material 2, all *P* < 0.05). While combining other renal biomarkers and mFRI increase the AUCs, there were no significant differences between these panels and mFRI alone.

### Prediction of composite outcome of hospital mortality or receipt of RRT

Thirty-three patients (3.3%) met the composite outcome of hospital mortality or receipt of RRT. Patients with composite outcomes displayed lower mFRI values and higher levels of renal biomarkers (Fig. S5 in Supplementary Material 6 and Table S1 in Supplementary Material 2, all *P* < 0.01). Regarding cytokines, patients with composite outcome demonstrated significantly higher levels of TNF, IL-2R and IL-8 (all *P* < 0.05). However, no significant differences were observed in the levels of IL-1β, IL-6 and IL-10 between the two groups.

The mFRI, with an AUC of 0.85 (95% CI 0.82–0.87), also exhibited significant predictive ability for the composite outcome. When comparing the AUCs head-to-head, the predictive performance of mFRI was comparable to sNGAL(AUC 0.79, 95%CI 0.76–0.81), sCys C (AUC 0.77, 95%CI 0.74–0.79), uACR (AUC 0.81, 95%CI 0.79–0.84), uNGAL/uCr (AUC 0.83, 95%CI 0.80–0.85) and uNAG/uCr (AUC 0.83, 95%CI 0.80–0.85,Fig. 2 and Table S4 in Supplementary Material 2). The optimal cutoff value of mFRI was 0.10 mL/(mg·kg)/2 h for composite outcome, with sensitivity of 79.66% (95% CI 67.2–89.0) and specificity of 81.97% (95% CI 79.4–84.4).

We also examined the predictive value of combined biomarkers for composite outcome. Among the evaluated panels, the combination of mFRI and uNAG/uCr demonstrated the highest AUC values (AUC 0.89, 95%CI 0.87–0.91). The addition of renal biomarkers significantly improved the AUC of mFRI except mFRI plus sCys C (Fig. S3 in Supplementary Material 4 and Table S5 in Supplementary Material 2).

### Associations between mFRI and adverse renal outcomes

Multivariable logistic models were utilized to evaluate the association between biomarkers and adverse renal outcomes. The mFRI showed significant associations with AKI progression, progression to stage 3, and composite outcomes after adjusting age, gender, BMI, diabetes mellitus, hypertension, CAD, cerebrovascular disease, preoperative diuretic exposure, baseline eGFR, surgical type, cardiopulmonary bypass used, CVP, AKI stage at enrollment and SOFA score (Table 4, all *P* < 0.05).

**Table 4:** Associations between biomarkers and adverse renal outcomes

	AKI progression				AKI progression to stage 3				Composite outcome			
Biomarkers	Unadjusted OR* (95% CI)	*P* value	Adjusted OR* (95% CI)	*P* value	Unadjusted OR* (95% CI)	*P* value	Adjusted OR* (95% CI)	*P* value	Unadjusted OR* (95% CI)	*P* value	Adjusted OR* (95% CI)	*P* value
Proximal tubular function												
mFRI, mL/(mg·kg)/2 h	0.25(0.19–0.31)	< 0.01	0.28(0.21–0.37)	< 0.01	0.18(0.13–0.26)	< 0.01	0.26(0.17–0.39)	< 0.01	0.3(0.21–0.43)	< 0.01	0.48(0.27–0.86)	0.01
**Traditional biomarker**												
uACR, ug/mg	1.9(1.63–2.22)	< 0.01	1.61(1.34–1.94)	< 0.01	2.4(1.93–2.98)	< 0.01	2.26(1.69–3.03)	< 0.01	2.36(1.82–3.06)	< 0.01	1.96(1.3–2.95)	< 0.01
**GFR biomarker**												
sCys C, mg/L	19.63(11.11–34.66)	< 0.01	40.59(16.68–98.77)	< 0.01	25.19(11.19–56.74)	< 0.01	20.78(5.69–75.93)	< 0.01	17.22(6.38–46.5)	< 0.01	5.9(0.99–35.3)	0.05
**Kidney injury biomarkers**												
sNGAL, ug/L	7.23(4.89–10.68)	< 0.01	6.95(4.2–11.5)	< 0.01	7.75(4.51–13.33)	< 0.01	8.4(3.76–18.74)	< 0.01	6.85(3.52–13.33)	< 0.01	4.04(1.3-12.57)	0.02
uNGAL/uCr, ng/mg	2.11(1.79–2.48)	< 0.01	1.75(1.43–2.14)	< 0.01	3.01(2.4–3.78)	< 0.01	2.38(1.78–3.18)	< 0.01	2.66(2.08–3.4)	< 0.01	1.66(1.17–2.36)	< 0.01
uNAG/uCr, U/mg	4.37(3.3–5.8)	< 0.01	3.29(2.37–4.56)	< 0.01	6.46(4.32–9.66)	< 0.01	4.05(2.46–6.65)	< 0.01	5.28(3.35–8.31)	< 0.01	2.27(1.29–3.98)	< 0.01
**Circulating inflammatory biomarkers**												
TNF, pg/mL	1.79(1.32–2.42)	< 0.01	1.43(0.97–2.1)	0.07	1.37(0.86–2.19)	0.19	0.93(0.51–1.72)	0.82	1.83(1.06–3.15)	0.03	1.1(0.48–2.52)	0.82
IL-1β, pg/mL	1.07(0.62–1.83)	0.81	1.42(0.78–2.56)	0.25	0.98(0.42–2.31)	0.96	1.65(0.66–4.15)	0.28	0.22(0.02–2.05)	0.18	0.34(0.02–5.48)	0.45
IL-2R, U/mL	2.93(1.95–4.41)	< 0.01	1.35(0.83–2.2)	0.23	2.17(1.18–3.99)	0.01	0.52(0.24–1.13)	0.1	8.62(3.84–19.35)	< 0.01	3.12(0.97–10.03)	0.06
IL-6, pg/mL	1.06(0.85–1.33)	0.58	1.11(0.85–1.44)	0.45	0.74(0.53–1.02)	0.06	0.71(0.46–1.09)	0.11	0.77(0.51–1.18)	0.23	0.61(0.32–1.15)	0.13
IL-8, pg/mL	1.91(1.48–2.45)	< 0.01	1.55(1.12–2.12)	0.01	1.56(1.08–2.24)	0.02	0.93(0.58–1.49)	0.75	2.24(1.47–3.4)	< 0.01	0.86(0.46–1.61)	0.64
IL-10, pg/mL	2.06(1.7–2.48)	< 0.01	1.83(1.47–2.27)	< 0.01	1.95(1.5–2.53)	< 0.01	1.61(1.17–2.2)	0.003	2.4(1.75–3.31)	< 0.01	1.83(1.19–2.8)	0.006

The multivariable models were adjusted for age, gender, BMI, diabetes mellitus, hypertension, CAD, cerebrovascular disease, preoperative diuretic exposure, baseline eGFR, surgical type, cardiopulmonary bypass used, CVP, AKI stage at enrollment and SOFA score. *, odds ratio for natural log-continuous biomarkers AKI, acute kidney injury, BMI, body mass index, CAD, coronary artery disease, CI, confidence interval, CVP, central venous pressure, eGFR, estimated glomerular filtration rate, IL-1β, interleukin-1β, IL-2R, interleukin-2 receptor, IL-6, interleukin-6, IL-8, interleukin-8, IL-10, interleukin-10, mFRI, modified furosemide responsiveness index, OR, odds ratio, SOFA score, sequential organ failure assessment score, sCysC, serum cystatin C, sNGAL, serum neutrophil gelatinase-associated lipocalin, TNF, tumor necrosis factor, uACR, urinary albumin/creatinine ratio, uCr, urinary creatinine, uNAG, urinary N-acetyl-β-D-glucosaminidase, uNGAL, urinary neutrophil gelatinase-associated lipocalin

### Contribution of renal biomarkers panels to the clinical model for adverse renal outcomes

Based on the superior predictive performance of the combination of mFRI and uNAG/uCr in predicting AKI progression and composite outcome, as well as the highest AUC of the panel comprising mFRI and uNGAL/uCr in predicting AKI progression to stage 3, these two panels were selected for further analysis. Incorporating mFRI alone or the two panels (mFRI and uNAG/uCr or mFRI and uNGAL/uCr) into the clinical model significantly improved the predictive ability for AKI progression, AKI progression to stage 3, and the composite outcome, as demonstrated by the C index, NRI, and IDI (Table S6 in Supplementary Material 2, all *P* < 0.01).

## Discussion

To the best of our knowledge, this study is the first comprehensive examination to compare the predictive performance of mFRI and novel biomarkers measured at AKI diagnosis for adverse renal outcomes in a large cohort of cardiac surgical patients with initial mild and severe AKI. Our findings indicated that mFRI exhibited superiority or non-inferiority to renal biomarkers and inflammation cytokines in its ability to predict AKI progression and prognosis. Furthermore, the combination of a functional biomarker (mFRI) and a urinary injury biomarker (uNAG or uNGAL) resulted in a improvement in the prediction of adverse outcomes than either individual biomarker. Moreover, the panel into clinical model significantly enhanced its predictive capacity.

Furosemide is commonly prescribed for fluid management in critical ill patients. It acts by inhibiting sodium reabsorption at the ascending limb of the loop of Henle, leading to increased natriuresis and urine output [12, 13]. However, in the presence of AKI, a diminished diuretic response to furosemide is frequently observed and is associated with the severity of AKI [14]. Therefore, assessing the diuretic response can serve as a cost-effective and simple method to evaluate renal tubular function during AKI. The furosemide stress test (FST), introduced by Koyner et al. in 2013, is a standardized method for assessing diuretic response through the administration of high-dose intravenous furosemide [15]. The FST has demonstrated significant discriminatory power in predicting AKI progression to stage 3 in patients with early AKI [15], with subsequent studies confirming its predictive capability in identifying AKI progression [16, 17]. However, concerns regarding the potential adverse effects of high-dose furosemide and hypovolemia may limit its widespread use. Two preliminary studies have simplified the FST as furosemide responsiveness (FR), quantified by 2-hour urine output following different furosemide doses. These studies have shown effective discrimination for AKI occurrence in pediatric patients and AKI progression in critically ill adults [18, 19]. In our previous work, we introduced mFRI as a new biomarker to quantify diuretic response by calculating the ratio of 2-hour urine output to nonstandardized furosemide dose and body weight. An inverse association between mFRI and the risk of AKI progression was observed in patients with early AKI following cardiac surgery in two independent cohorts [11]. The mFRI presents several advantages: (1) bedside operability, (2) rapid results, (3) cost-effectiveness, (4) universal accessibility, and (5) integrated testing and treatment features. These features establish it as a cost-effective and universally applicable tool for the early identification of AKI progression. Our current findings confirmed the superiority or non-inferiority of mFRI compared to novel renal biomarkers and cytokines in predicting AKI progression and prognosis.

sCys C, a small protein synthesized uniformly by all nucleated cells, undergoes free filtration at the glomerulus and complete reabsorption and catabolism by proximal tubular cells without tubular secretion [20]. sCys C has been recognized as a sensitive and specific biomarker for estimating GFR [21, 22]. While sCysC has been linked to AKI and adverse outcomes, its predictive accuracy for AKI progression shows significant variability [5, 7, 23]. Our study revealed that sCys C showed good predictive capabilities for AKI progression, progression to stage 3, and composite outcome. However, the AUCs for adverse renal outcomes were slightly lower than those of mFRI, with no statistical significance.

Albumin, which can pass through the filtration barrier in small amounts, is typically reabsorbed by the proximal tubule. Elevated urinary albumin levels indicate increased permeability of the glomerular basal membrane due to injury, making it a valuable diagnostic indicator for renal diseases, including AKI [24–26]. In this study, the AUC of urinary albumin/creatinine ratio (uACR) was significantly lower than that of mFRI in predicting AKI progression. However, the differences were not statistically significant when considering progression to stage 3 AKI and the composite outcome.

NGAL is a protein that belongs to the lipocalin family and is expressed in various tissues, including the kidneys [27]. NGAL is markedly induced in injured renal tubular cells in response to injury [27]. Previous studies revealed that both sNGAL and uNGAL are associated with AKI occurrence and adverse outcomes among adults undergoing cardiac surgery [28]. Furthermore, sNGAL measured at AKI diagnosis could identify patients at higher risk for AKI progression and adverse outcomes [8]. NAG is an enzyme predominantly localized within the lysosomes of renal tubular cells. Elevated levels of uNAG are recognized as a sensitive biomarker for detecting renal tubular damage, which may be attributed to various conditions, including AKI and chronic kidney disease [20]. Our results indicate that mFRI is superior or comparable in predicting AKI progression and prognosis compared to the kidney injury biomarkers.

Inflammation plays a crucial role in the pathophysiology of AKI. Elevated levels of cytokines, such as IL-6, IL-8, IL-10, and TNF, have been linked to an increased risk of AKI in patients across various clinical settings, including cardiac surgery [29, 30], sepsis [31], and acute lung injury [32]. Studies have reported an association between plasma IL-8 levels and the progression of AKI in adult and pediatric patients following cardiac surgery [5]. In this study, TNF, IL-2R, IL-8, and IL-10 showed limited predictive value for AKI progression (AUC range, 0.60–0.67) and composite outcome (AUC range, 0.63–0.75). This discrepancy may be attributed to the ability of the mFRI, derived from urine, to accurately detect local tubular dysfunction, in contrast to systemic inflammation biomarkers that lack specificity for renal injury process due to potential confounding factors such as multiorgan dysfunction.

Recent research has highlighted the improved predictive potential for AKI diagnosis and prognosis by combining damage and functional biomarkers [33, 34]. In our study, mFRI was identified as a novel biomarker of tubular function, and its utility in combination with renal biomarkers was evaluated for predicting adverse renal outcomes. The combination of mFRI and uNAG/uCr yielded the highest AUC values for predicting AKI progression and composite outcome, with a noteworthy AUC observed for the panel comprising mFRI and uNGAL/uCr in predicting AKI progression to stage 3. Incorporating the two panels (mFRI and uNAG/uCr or mFRI and uNGAL/uCr) into the clinical model improved the predictive ability for adverse renal outcomes. This could be attributed to the complementary nature of functional and tubular damage biomarkers, which capture distinct aspects of nephron damage. Our findings are consistent with the recommendations of the Acute Disease Quality Initiative (ADQI) Consensus Conference, which advocate for the use of a combination of damage and functional biomarkers to identify high-risk patient groups, enhance care processes, and aid in the management of AKI [35]. While the combination of mFRI with damage biomarker enhances discriminatory capacity, the rise in AUC is relatively modest. Future studies with larger cohorts encompassing diverse etiologies or AKI risk factors are necessary.

Our study has several limitations. Firstly, it was conducted as a single-center observational study, specifically focusing on patients with cardiac surgery-associated AKI. Secondly, the prescription of furosemide was individually determined based on the patient’s condition, resulting in vague indications and a lack of standardization in the administered dose. This nonstandardized approach may have compromised the discriminatory ability of mFRI. Future studies should consider implementing a predefined furosemide prescription protocol to address this limitation. Thirdly, certain biomarkers, like [TIMP-2]·[IGFBP7], was not evaluated in this study. Lastly, our study only assessed biomarkers at the time of AKI diagnosis, warranting further investigation to compare the performance of mFRI and biomarkers kinetics for AKI progression.

## Conclusions

As a rapid, cost-effective and easily accessible biomarker, mFRI exhibited superiority or non-inferiority to renal biomarkers in its ability to predict AKI progression and prognosis in cardiac surgical patients with mild to moderate AKI. Furthermore, the combination of a functional biomarker (mFRI) and a urinary injury biomarker (uNAG or uNGAL) resulted in an improvement in the prediction of adverse renal outcomes than either individual biomarker.

## Electronic supplementary material

Below is the link to the electronic supplementary material.

Supplementary Material 1

Supplementary Material 2

Supplementary Material 3

Supplementary Material 4

Supplementary Material 5

Supplementary Material 6

## Data Availability

All data generated or analyzed during this study are included in this published article [and its supplementary information files].

## Abbreviations

AKI: Acute kidney injury
AUC: Area under the curve
CKD: Chronic kidney disease
CVP: Central venous pressure
eGFR: Estimated glomerular filtration rate
GFR: Glomerular filtration rate
IL-1β: Interleukin-1β
IL-2R: Interleukin-2 receptor
IL-6: Interleukin-6
IL-8: Interleukin-8
IL-10: Interleukin-10
LR+: Positive likelihood ratio
LR-: Negative likelihood ratio
mFRI: Modified furosemide responsiveness index
NPV: Negative predictive value
PPV: Positive predictive value
RRT: +Renal replacement therapy
SEM: Standard error of mean
sCysC: Serum cystatin C
sNGAL: Serum neutrophil gelatinase-associated lipocalin
SOFA score: Sequential organ failure assessment score
TNF: Tumor necrosis factor
uACR: Urinary albumin/creatinine ratio
uCr: Urinary creatinine
uNAG: Urinary N-acetyl-β-D-glycosaminidase
uNGAL: Urinary neutrophil gelatinase-associated lipocalin
