# Supplementary figures and images for: Modified furosemide responsiveness index and biomarkers for AKI progression and prognosis: a prospective observational study
Source: Ann Intensive Care. 2024 Oct 8;14:156. doi: 10.1186/s13613-024-01387-y (PMC11461418; doi:10.1186/s13613-024-01387-y)

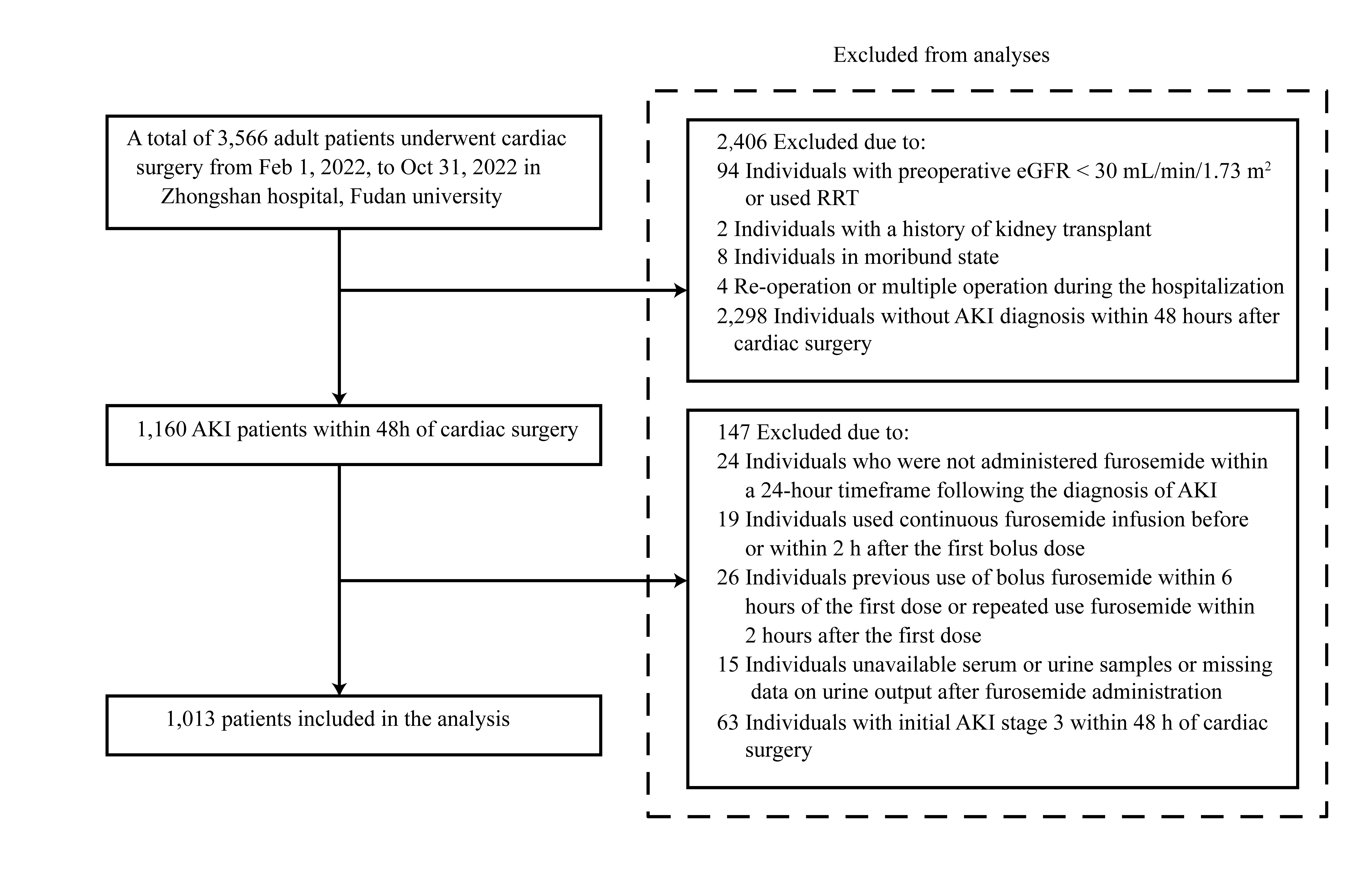

Supplement: Supplementary file 1 — Supplementary Material 1 [file 13613_2024_1387_MOESM1_ESM.tif]

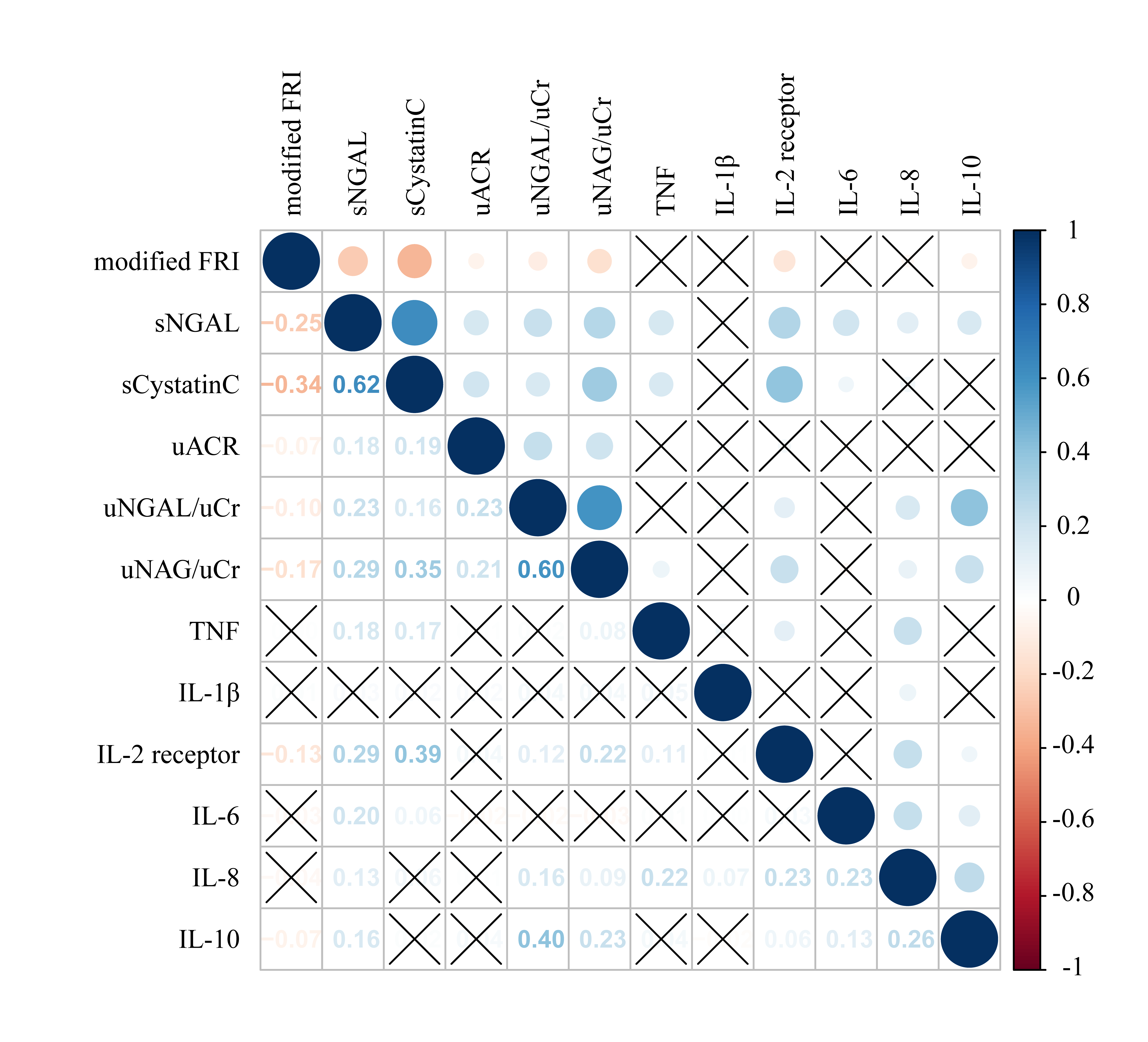

Supplement: Supplementary file 3 — Supplementary Material 3 [file 13613_2024_1387_MOESM3_ESM.tif]

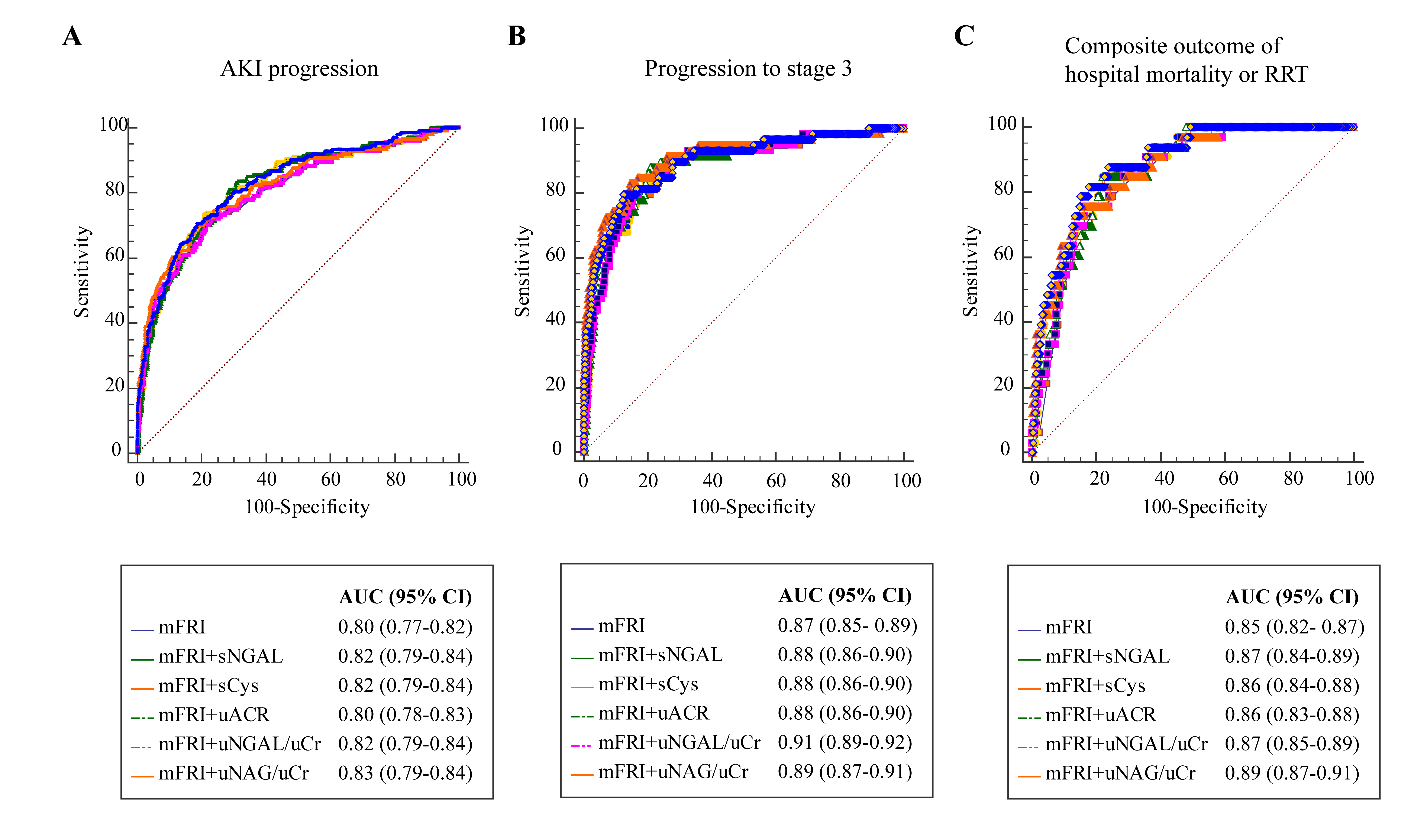

Supplement: Supplementary file 4 — Supplementary Material 4 [file 13613_2024_1387_MOESM4_ESM.tif]

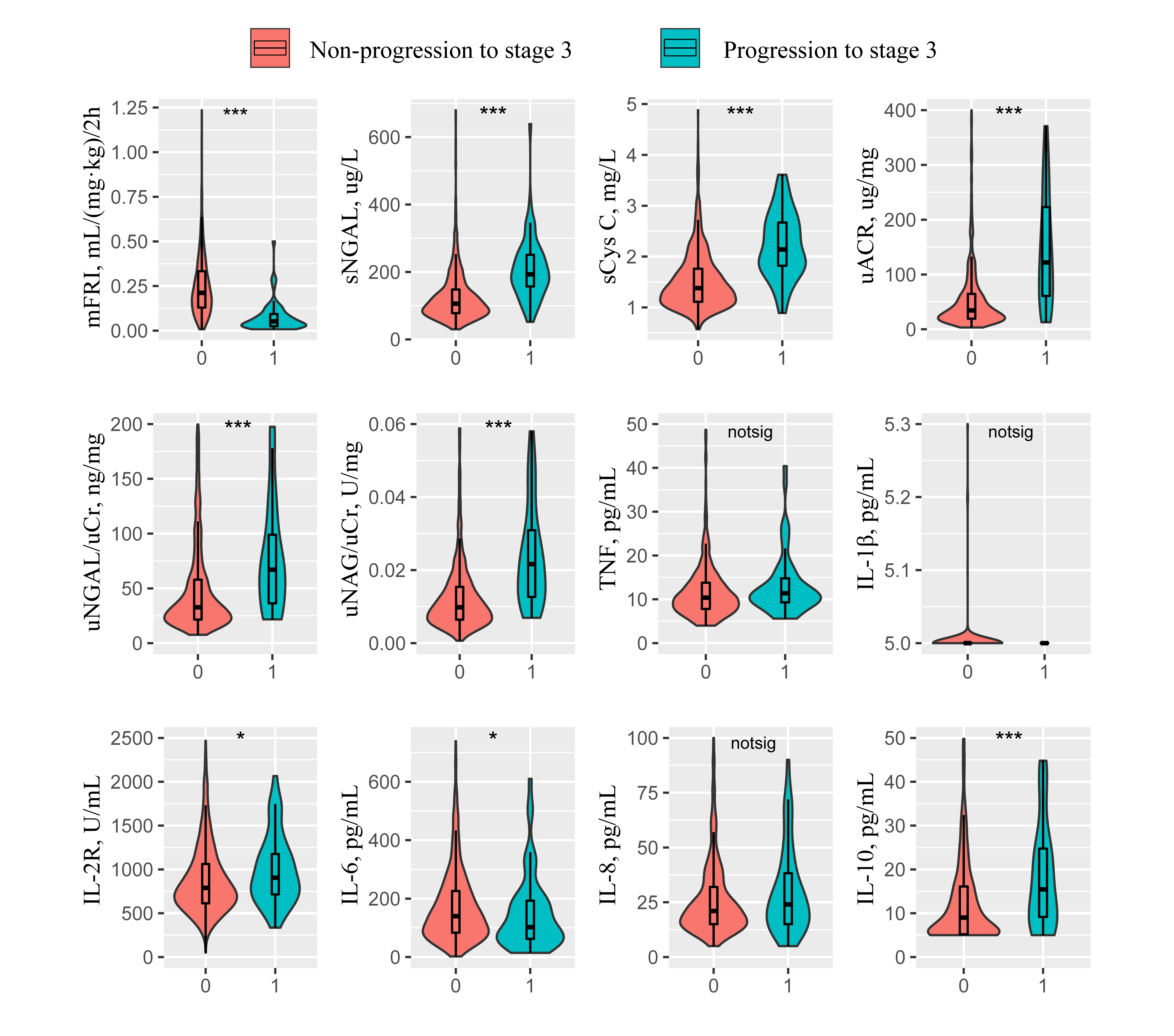

Supplement: Supplementary file 5 — Supplementary Material 5 [file 13613_2024_1387_MOESM5_ESM.tif]

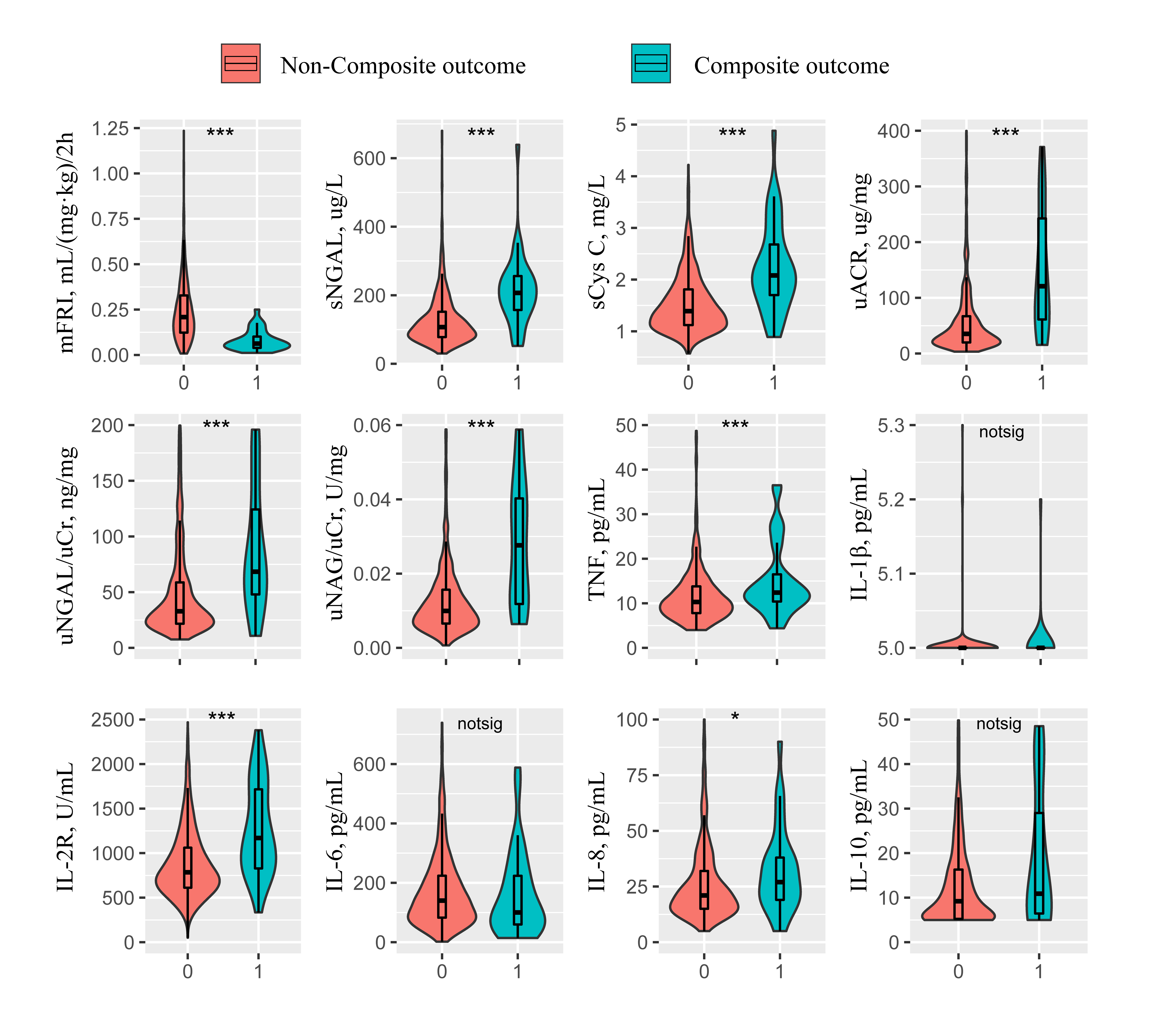

Supplement: Supplementary file 6 — Supplementary Material 6 [file 13613_2024_1387_MOESM6_ESM.tif]
