# Supplementary material for: Modified furosemide responsiveness index and biomarkers for AKI progression and prognosis: a prospective observational study
Source: Ann Intensive Care. 2024 Oct 8;14:156. doi: 10.1186/s13613-024-01387-y (PMC11461418; doi:10.1186/s13613-024-01387-y)
Supplement: Supplementary file 2 — Supplementary Material 2 [file 13613_2024_1387_MOESM2_ESM.docx]

**Supplementary Appendix**

**Table S1. Biomarkers concentrations stratified by adverse renal outcomes**

**Table S2. Predictive performance of biomarkers for AKI progression to stage 3**

**Table S3. Predictive performance of combined mFRI and renal biomarkers for AKI progression to stage 3**

**Table S4. Predictive performance of biomarkers for composite outcome of hospital mortality or RRT**

**Table S5. Predictive performance of combined mFRI and renal biomarkers for composite outcome of hospital mortality or RRT**

**Table S6. Predictive value of biomarker panels added to baseline clinical model**

**Table S1. Biomarkers concentrations stratified by adverse renal outcomes**

|  | **Biomarker concentration, median [IQR]** | | | | | | | | |
| --- | --- | --- | --- | --- | --- | --- | --- | --- | --- |
| **Biomarkers** | **AKI progression** | | | **AKI progression to stage 3** | | | **Composite outcome** | | |
|  | **No (n= 859)** | **Yes (n= 154)** | ***P* value** | **No（n=954）** | **Yes（n=59）** | ***P* value** | **No（n=980）** | **Yes（n=33）** | ***P* value** |
| **Tubular function biomarker** |  |  |  |  |  |  |  |  |  |
| mFRI, mL/(mg·kg)/2h | 0.22[0.14,0.34] | 0.08[0.04,0.16] | <0.01 | 0.21[0.13,0.33] | 0.05[0.03,0.1] | <0.01 | 0.21[0.12,0.33] | 0.06[0.04,0.11] | <0.01 |
| **Traditional biomarker** |  |  |  |  |  |  |  |  |  |
| urinary albumin, mg/L | 34.8[18.3,64.2] | 51.8[28.93,109.78] | <0.01 | 35.15[19.18,65.33] | 80.8[32.4,148] | <0.01 | 35.5[19.3,68.05] | 66.7[26.85,131.25] | 0.01 |
| urinary creatinine, mg/dl | 101.27[59.38,152.02] | 77.62[48.48,108.46] | <0.01 | 100.54[59.32,150.24] | 52.22[32.69,85.96] | <0.01 | 99.92[58.36,148.75] | 46.24[30.33,63.55] | <0.01 |
| uACR, ug/mg | 34.7[19.3,66.5] | 80.75[33.25,190.53] | <0.01 | 36.05[20.08,69.83] | 170.6[88,301.6] | <0.01 | 37.05[20.3,74.73] | 173.5[64.75,358.35] | <0.01 |
| **GFR biomarker** |  |  |  |  |  |  |  |  |  |
| sCys C, mg/L | 1.33[1.1,1.69] | 1.97[1.61,2.39] | <0.01 | 1.38[1.11,1.76] | 2.14[1.82,2.68] | <0.01 | 1.39[1.12,1.81] | 2.08[1.69,2.8] | <0.01 |
| **Kidney injury biomarkers** |  |  |  |  |  |  |  |  |  |
| sNGAL, ug/L | 102[77,139] | 174[125,233.25] | <0.01 | 106[78,148] | 193[156,256] | <0.01 | 107[78,152] | 207[156,257] | <0.01 |
| uNGAL, ug/L | 30[30,31] | 42.45[30,101.5] | <0.01 | 30[30,33.65] | 78.4[30,256.9] | <0.01 | 30[30,34.7] | 68.7[30,277.55] | <0.01 |
| uNGAL/uCr, ng/mg | 33.55[21.62,64.38] | 61.72[31.53,196.32] | <0.01 | 34.32[21.96,65.75] | 151.48[61.78,1088] | <0.01 | 34.99[22.17,68.87] | 185.1[66.62,994.65] | <0.01 |
| uNAG, U/L | 9.5[5.3,15.3] | 14.05[8.75,21.73] | <0.01 | 10[5.6,16] | 13.6[8.5,23.7] | <0.01 | 10.1[5.7,16] | 14.2[6,21.9] | 0.25 |
| uNAG/uCr, U/mg | 0.01[0.01,0.01] | 0.02[0.01,0.03] | <0.01 | 0.01[0.01,0.02] | 0.02[0.01,0.05] | <0.01 | 0.01[0.01,0.02] | 0.03[0.01,0.05] | <0.01 |
| **Circulating inflammatory biomarkers** |  |  |  |  |  |  |  |  |  |
| TNF, pg/mL | 10.3[7.7,13.7] | 12.2[9.3,16] | <0.01 | 10.5[7.8,14] | 11.4[9.25,14.8] | 0.10 | 10.4[7.8,13.9] | 12.4[10.3,16.7] | 0.09 |
| IL-1β, pg/mL | 5[5,5] | 5[5,5] | 0.70 | 5[5,5] | 5[5,5] | 0.90 | 5[5,5] | 5[5,5] | 0.87 |
| IL-2R, U/mL | 775[608,1045.5] | 953[715,1300] | <0.01 | 792[615.75,1065.5] | 906[714,1208.84] | 0.01 | 788.5[614.25,1065] | 1192.67[828,1805] | <0.01 |
| IL-6, pg/mL | 140[83.5,224.75] | 144[75.6,276] | 0.72 | 142[83.88,228] | 108[61.25,202] | 0.05 | 141.5[82.93,227] | 103[58.05,240] | 0.50 |
| IL-8, pg/mL | 21[15,31] | 28[17,49] | <0.01 | 22[15,33.63] | 26[15,44.5] | 0.09 | 21[15,33] | 32[19,54] | <0.01 |
| IL-10, pg/mL | 9.2[5.23,16.7] | 17.3[8.1,36.3] | <0.01 | 9.7[5.4,17.83] | 19.5[9.7,39.35] | <0.01 | 9.9[5.5,18.13] | 23.9[8,88.65] | <0.01 |

AKI, acute kidney injury; GFR, glomerular filtration rate; IL-1β, interleukin-1β; IL-2R, interleukin-2 receptor; IL-6, interleukin-6; IL-8, interleukin-8; IL-10, interleukin-10; IQR, interquartile range; mFRI, modified furosemide responsiveness index; SEM, standard error of mean; sCysC, serum cystatin C; sNGAL, serum neutrophil gelatinase-associated lipocalin; TNF, tumor necrosis factor; uACR, urinary albumin/creatinine ratio; uCr, urinary creatinine; uNAG, urinary N-acetyl-β-D-glucosaminidase; uNGAL, urinary neutrophil gelatinase-associated lipocalin.

**Table S2. Predictive performance of biomarkers for AKI progression to stage 3**

| **Biomarkers** | **AUC±SEM** | **95%CI** | ***P* Value** | ***P* Value Compared With mFRI** | **Cutoff** | **Sensitivity (95%CI)** | **Specificity (95%CI)** | **LR+ (95%CI)** | **LR- (95%CI)** | **PPV (95%CI)** | **NPV (95%CI)** |
| --- | --- | --- | --- | --- | --- | --- | --- | --- | --- | --- | --- |
| **Tubular function biomarker** |  |  |  |  |  |  |  |  |  |  |  |
| mFRI, mL/(mg·kg)/2h | 0.87±0.03 | 0.85- 0.89 | <0.01 | - | 0.10 | 79.66(67.2 - 89.0) | 81.97(79.4 - 84.4) | 4.42 (3.7 - 5.3) | 0.25(0.1 - 0.4) | 21.5(16.2 - 27.5) | 98.5(97.4 - 99.2) |
| **Traditional biomarker** |  |  |  |  |  |  |  |  |  |  |  |
| uACR, ug/mg | 0.82±0.03 | 0.79- 0.84 | <0.01 | 0.15 | 87.8 | 76.27(63.4 - 86.4) | 80.53(77.9 - 83.0) | 3.92(3.2 - 4.7) | 0.29(0.2 - 0.5) | 19.6(14.6 - 25.3) | 98.2(97.0 - 99.0) |
| **GFR biomarker** |  |  |  |  |  |  |  |  |  |  |  |
| sCys C, mg/L | 0.81±0.03 | 0.79- 0.84 | <0.01 | 0.049 | 1.76 | 81.36(69.1 - 90.3) | 75.26(72.4 - 78.0) | 3.29(2.8 - 3.9) | 0.25(0.1 - 0.4) | 17(12.8 - 21.9) | 98.5(97.3 - 99.2) |
| **Kidney injury biomarkers** |  |  |  |  |  |  |  |  |  |  |  |
| sNGAL, ug/L | 0.80±0.03 | 0.78- 0.83 | <0.01 | 0.02 | 150 | 77.97(65.3 - 87.7) | 75.66(72.8 - 78.4) | 3.2(2.7 - 3.8) | 0.29(0.2 - 0.5) | 16.6(12.4 - 21.5) | 98.2(97.0 - 99.0) |
| uNGAL/uCr, ng/mg | 0.82±0.03 | 0.79- 0.84 | <0.01 | 0.08 | 61.3 | 77.97(65.3 - 87.7) | 73.31(70.4 - 76.1) | 2.92(2.5 - 3.5) | 0.3(0.2 - 0.5) | 15.4(11.5 - 20.0) | 98.2(96.9 - 99.0) |
| uNAG/uCr, U/mg | 0.84±0.03 | 0.81- 0.85 | <0.01 | 0.18 | 0.02 | 67.8(54.4 - 79.4) | 83.25(80.7 - 85.6) | 4.05(3.2 - 5.1) | 0.39(0.3 - 0.6) | 20.1(14.8 - 26.3) | 97.7(96.4 - 98.6) |
| **Circulating inflammatory biomarkers** |  |  |  |  |  |  |  |  |  |  |  |
| TNF, pg/mL | 0.56±0. 04 | 0.53-0.60 | 0.10 | <0.001 | 8.7 | 82.46(70.1 - 91.3) | 34.5(31.5 - 37.6) | 1.26(1.1 - 1.4) | 0.51 (0.3 - 0.9) | 7.1(5.2 - 9.3) | 97(94.6 - 98.6) |
| IL-1β, pg/mL | 0.50±0.04 | 0.47- 0.54 | 0.91 | <0.001 | 8.8 | 96.49(87.9 - 99.6) | 8.49(6.8 - 10.5) | 1.05(1.0 - 1.1) | 0.41(0.1 - 1.6) | 6(4.5 - 7.7) | 97.6(91.5 - 99.7) |
| IL-2R, U/mL | 0.60±0.04 | 0.57- 0.63 | 0.01 | <0.001 | 878 | 57.89(44.1 - 70.9) | 60.19(57.0 - 63.3) | 1.45(1.1 - 1.8) | 0.7(0.5 - 1.0) | 8.1(5.6 - 11.2) | 95.9(94.0 - 97.4) |
| IL-6, pg/mL | 0.58±0.04 | 0.55- 0.61 | 0.05 | <0.001 | 88.8 | 45.61(32.4 - 59.3) | 73.57(70.6 - 76.4) | 1.73(1.3 - 2.3) | 0.74(0.6 - 0.9) | 9.5(6.3 - 13.5) | 95.7(94.0 - 97.1) |
| IL-8, pg/mL | 0.57±0.04 | 0.54- 0.60 | 0.10 | <0.001 | 26 | 49.12(35.6 - 62.7) | 65.07(61.9 - 68.1) | 1.41(1.1 - 1.9) | 0.78(0.6 - 1.0) | 7.8(5.3 - 11.1) | 95.5(93.6 - 97.0) |
| IL-10, pg/mL | 0.68±0.04 | 0.65- 0.71 | <0.01 | <0.001 | 16.9 | 56.14(42.4 - 69.3) | 73.04(70.1 - 75.8) | 2.08(1.6 - 2.7) | 0.6(0.4 - 0.8) | 11.2(7.8 - 15.4) | 96.5(94.9 - 97.7) |

AKI, acute kidney injury; AUC, area under the curve; CI, confidence interval; GFR, glomerular filtration rate; IL-1β, interleukin-1β; IL-2R, interleukin-2 receptor; IL-6, interleukin-6; IL-8, interleukin-8; IL-10, interleukin-10; mFRI, modified furosemide responsiveness index; SEM, standard error of mean; sCysC, serum cystatin C; sNGAL, serum neutrophil gelatinase-associated lipocalin; TNF, tumor necrosis factor; uACR, urinary albumin/creatinine ratio; uCr, urinary creatinine; uNAG, urinary N-acetyl-β-D-glucosaminidase; uNGAL, urinary neutrophil gelatinase-associated lipocalin; LR+, Positive likelihood ratio; LR-, negative likelihood ratio; PPV, Positive predictive value; NPV, Negative predictive value.

**Table S3. Predictive performance of combined mFRI and renal biomarkers for AKI progression to stage 3**

| **Biomarkers** | **AUC±SEM** | **95%CI** | ***P* Value** | ***P* Value Compared With mFRI** | **Cutoff** | **Sensitivity (95%CI)** | **Specificity (95%CI)** | **LR+ (95%CI)** | **LR- (95%CI)** | **PPV (95%CI)** | **NPV (95%CI)** |
| --- | --- | --- | --- | --- | --- | --- | --- | --- | --- | --- | --- |
| **Tubular function biomarker** |  |  |  |  |  |  |  |  |  |  |  |
| mFRI | 0.87±0.03 | 0.85- 0.89 | <0.01 | - | 0.10 | 79.66(67.2 - 89.0) | 81.97(79.4 - 84.4) | 4.42 (3.7 - 5.3) | 0.25(0.1 - 0.4) | 21.5(16.2 - 27.5) | 98.5(97.4 - 99.2) |
| **Biomarker combination** |  |  |  |  |  |  |  |  |  |  |  |
| mFRI + uACR | 0.88±0.02 | 0.86-0.90 | <0.01 | 0.12 | 0.09 | 81.36 (69.1 - 90.3) | 82.84 (80.3 - 85.2) | 4.74 (3.9 - 5.7) | 0.23 (0.1 - 0.4) | 22.7 (17.3 - 29.0) | 98.6 (97.5 - 99.3) |
| mFRI+ sCys C | 0.88±0.02 | 0.86-0.90 | <0.01 | 0.17 | 0.07 | 88.14 (77.1 - 95.1) | 79.16 (76.4 - 81.7) | 4.23 (3.6 - 4.9) | 0.15 (0.07 - 0.3) | 20.8 (15.9 - 26.4) | 99.1 (98.1 - 99.6) |
| mFRI+sNGAL | 0.88±0.02 | 0.86-0.90 | <0.01 | 0.11 | 0.06 | 88.14 (77.1 - 95.1) | 76.29 (73.5 - 79.0) | 3.72 (3.2 - 4.3) | 0.16 (0.08 - 0.3) | 18.8 (14.3 - 23.9) | 99 (98.0 - 99.6) |
| mFRI+uNGAL/uCr | 0.91±0.02 | 0.89-0.92 | <0.01 | <0.01 | 0.08 | 83.05 (71.0 - 91.6) | 85.23 (82.8 - 87.4) | 5.62 (4.6 - 6.8) | 0.2 (0.1 - 0.4) | 25.9 (19.8 - 32.8) | 98.8 (97.8 - 99.4) |
| mFRI+uNAG/uCr | 0.89±0.02 | 0.87-0.91 | <0.01 | <0.01 | 0.09 | 79.66 (67.2 - 89.0) | 87.46 (85.2 - 89.5) | 6.35 (5.1 - 7.9) | 0.23 (0.1 - 0.4) | 28.3 (21.6 - 35.8) | 98.6 (97.5 - 99.3) |

AKI, acute kidney injury; AUC, area under the curve; CI, confidence interval; mFRI, modified furosemide responsiveness index; SEM, standard error of mean; sCysC, serum cystatin C; sNGAL, serum neutrophil gelatinase-associated lipocalin; uACR, urinary albumin/creatinine ratio; uCr, urinary creatinine; uNAG, urinary N-acetyl-β-D-glucosaminidase; uNGAL, urinary neutrophil gelatinase-associated lipocalin; LR+, Positive likelihood ratio; LR-, negative likelihood ratio; PPV, Positive predictive value; NPV, Negative predictive value.

**Table S4. Predictive performance of biomarkers for composite outcome of hospital mortality or RRT**

| **Biomarkers** | **AUC±SEM** | **95%CI** | ***P* Value** | ***P* Value Compared With mFRI** | **Cutoff** | **Sensitivity (95%CI)** | **Specificity (95%CI)** | **LR+ (95%CI)** | **LR- (95%CI)** | **PPV (95%CI)** | **NPV (95%CI)** |
| --- | --- | --- | --- | --- | --- | --- | --- | --- | --- | --- | --- |
| **Tubular function biomarker** |  |  |  |  |  |  |  |  |  |  |  |
| mFRI, mL/(mg·kg)/2h | 0.85±0.03 | 0.82- 0.87 | <0.01 |  | 0.10 | 75.76(57.7 - 88.9) | 80.2(77.6 - 82.7) | 3.83(3.0 - 4.8) | 0.3(0.2 - 0.6) | 11.4(7.5 - 16.4) | 99(98.0 - 99.6) |
| **Traditional biomarker** |  |  |  |  |  |  |  |  |  |  |  |
| uACR, ug/mg | 0.81±0.04 | 0.79- 0.84 | <0.01 | 0.38 | 60.8 | 81.82(64.5 - 93.0) | 69.26(66.3 - 72.1) | 2.66(2.2 - 3.2) | 0.26(0.1 - 0.5) | 8.3(5.5 - 11.8) | 99.1(98.1 - 99.7) |
| **GFR biomarker** |  |  |  |  |  |  |  |  |  |  |  |
| sCys C, mg/L | 0.77±0.04 | 0.74- 0.79 | <0.01 | 0.08 | 1.67 | 78.79(61.1 - 91.0) | 68.95(65.9 - 71.8) | 2.54(2.1 - 3.1) | 0.31(0.2 - 0.6) | 7.9(5.2 - 11.4) | 99(97.9 - 99.6) |
| **Kidney injury biomarkers** |  |  |  |  |  |  |  |  |  |  |  |
| sNGAL, ug/L | 0.79±0.04 | 0.76- 0.81 | <0.01 | 0.18 | 149 | 81.82(64.5 - 93.0) | 74.05(71.2 - 76.8) | 3.15(2.6 - 3.8) | 0.25(0.1 - 0.5) | 9.6( 6.5 - 13.7) | 99.2(98.2 - 99.7) |
| uNGAL/uCr, ng/mg | 0.83±0.04 | 0.80- 0.85 | <0.01 | 0.60 | 63.6 | 81.82(64.5 - 93.0) | 73(70.1 - 75.8) | 3.03(2.5 - 3.7) | 0.25(0.1 - 0.5) | 9.3(6.2 - 13.3) | 99.2(98.2 - 99.7) |
| uNAG/uCr, U/mg | 0.83±0.04 | 0.80- 0.85 | <0.01 | 0.63 | 0.02 | 63.64(45.1 - 79.6) | 90.67(88.7 - 92.4) | 6.82(4.9 - 9.4) | 0.4(0.3 - 0.6) | 18.7(12.0 - 27.2) | 98.7(97.7 - 99.3) |
| **Circulating inflammatory biomarkers** |  |  |  |  |  |  |  |  |  |  |  |
| TNF, pg/mL | 0.63±0.05 | 0.60- 0.66 | <0.01 | <0.001 | 9.6 | 84.85(68.1 - 94.9) | 42.86(39.7 - 46.0) | 1.48(1.3 - 1.7) | 0.35(0.2 - 0.8) | 4.8(3.2 - 6.9) | 98.8(97.2 - 99.6) |
| IL-1β, pg/mL | 0.53±0.05 | 0.49- 0.56 | 0.61 | <0.001 | 5.5 | 90.91(75.7 - 98.1) | 19.36(16.9 - 22.0) | 1.13(1.0 - 1.3) | 0.47(0.2 - 1.4) | 3.7(2.5 - 5.3) | 98.4(95.5 - 99.7) |
| IL-2R, U/mL | 0.75±0.04 | 0.72- 0.78 | <0.01 | 0.07 | 813 | 87.88(71.8 - 96.6) | 53.62(50.4 - 56.8) | 1.89(1.6 - 2.2) | 0.23(0.09 - 0.6) | 6.1(4.1 - 8.6) | 99.2(98.0 - 99.8) |
| IL-6, pg/mL | 0.55±0.06 | 0.52- 0.58 | 0.29 | <0.001 | 63.3 | 33.33(18.0 - 51.8) | 85.51(83.1 - 87.7) | 2.3(1.4 - 3.8) | 0.78(0.6 - 1.0) | 7.3(3.7 - 12.7) | 97.4(96.1 - 98.4) |
| IL-8, pg/mL | 0.65±0.05 | 0.62- 0.68 | <0.01 | <0.001 | 23 | 69.7(51.3 - 84.4) | 56.63(53.4 - 59.8) | 1.61(1.3 - 2.0) | 0.54(0.3 - 0.9) | 5.2(3.3 - 7.7) | 98.2(96.7 - 99.1) |
| IL-10, pg/mL | 0.69±0.06 | 0.66- 0.72 | <0.01 | 0.01 | 33.5 | 48.48(30.8 - 66.5) | 90.58(88.6 - 92.3) | 5.15(3.4 - 7.7) | 0.57(0.4 - 0.8) | 15(8.8 - 23.1) | 98.1(97.0 - 98.9) |

AKI, acute kidney injury; AUC, area under the curve; CI, confidence interval; GFR, glomerular filtration rate; IL-1β, interleukin-1β; IL-2R, interleukin-2 receptor; IL-6, interleukin-6; IL-8, interleukin-8; IL-10, interleukin-10; mFRI, modified furosemide responsiveness index; SEM, standard error of mean; sCysC, serum cystatin C; sNGAL, serum neutrophil gelatinase-associated lipocalin; TNF, tumor necrosis factor; uACR, urinary albumin/creatinine ratio; uCr, urinary creatinine; uNAG, urinary N-acetyl-β-D-glucosaminidase; uNGAL, urinary neutrophil gelatinase-associated lipocalin; LR+, Positive likelihood ratio; LR-, negative likelihood ratio; PPV, Positive predictive value; NPV, Negative predictive value.

**Table S5. Predictive performance of combined mFRI and renal biomarkers for** **composite outcome of hospital mortality or RRT**

| **Biomarkers** | **AUC±SEM** | **95%CI** | ***P* Value** | ***P* Value Compared With mFRI** | **Cutoff** | **Sensitivity (95%CI)** | **Specificity (95%CI)** | **LR+ (95%CI)** | **LR- (95%CI)** | **PPV (95%CI)** | **NPV (95%CI)** |
| --- | --- | --- | --- | --- | --- | --- | --- | --- | --- | --- | --- |
| **Tubular function biomarker** |  |  |  |  |  |  |  |  |  |  |  |
| mFRI | 0.85±0.03 | 0.82- 0.87 | <0.01 |  | 0.10 | 75.76(57.7 - 88.9) | 80.2(77.6 - 82.7) | 3.83(3.0 - 4.8) | 0.3(0.2 - 0.6) | 11.4(7.5 - 16.4) | 99(98.0 - 99.6) |
| **Biomarker combination** |  |  |  |  |  |  |  |  |  |  |  |
| mFRI + uACR | 0.86±0.03 | 0.83-0.88 | <0.01 | 0.02 | 0.06 | 75.76 (57.7 - 88.9) | 83.4 (80.9 - 85.7) | 4.56 (3.6 - 5.8) | 0.29 (0.2 - 0.5) | 13.4 (8.8 - 19.1) | 99 (98.1 - 99.6) |
| mFRI+ sCys C | 0.86±0.02 | 0.84-0.88 | <0.01 | 0.15 | 0.04 | 84.85 (68.1 - 94.9) | 78.18 (75.5 - 80.7) | 3.89 (3.2 - 4.7) | 0.19 (0.09 - 0.4) | 11.6 (7.9 - 16.4) | 99.3 (98.5 - 99.8) |
| mFRI+sNGAL | 0.87±0.03 | 0.84-0.89 | <0.01 | 0.03 | 0.04 | 84.85 (68.1 - 94.9) | 78.05 (75.3 - 80.6) | 3.87 (3.2 - 4.7) | 0.19 (0.09 - 0.4) | 11.6 (7.8 - 16.3) | 99.3 (98.5 - 99.8) |
| mFRI+uNGAL/uCr | 0.87±0.03 | 0.85-0.89 | <0.01 | <0.001 | 0.05 | 75.76 (57.7 - 88.9) | 83.78 (81.3 - 86.0) | 4.67 (3.7 - 5.9) | 0.29 (0.2 - 0.5) | 13.7 (9.0 - 19.5) | 99 (98.1 - 99.6) |
| mFRI+uNAG/uCr | 0.89±0.02 | 0.87-0.91 | <0.01 | <0.001 | 0.03 | 87.88 (71.8 - 96.6) | 76.1 (73.3 - 78.7) | 3.68 (3.1 - 4.4) | 0.16 (0.06 - 0.4) | 11.1 (7.5 - 15.5) | 99.5 (98.6 - 99.9) |

AKI, acute kidney injury; AUC, area under the curve; CI, confidence interval; mFRI, modified furosemide responsiveness index; SEM, standard error of mean; sCysC, serum cystatin C; sNGAL, serum neutrophil gelatinase-associated lipocalin; uACR, urinary albumin/creatinine ratio; uCr, urinary creatinine; uNAG, urinary N-acetyl-β-D-glucosaminidase; uNGAL, urinary neutrophil gelatinase-associated lipocalin; LR+, Positive likelihood ratio; LR-, negative likelihood ratio; PPV, Positive predictive value; NPV, Negative predictive value.

**Table S6. Predictive value of biomarker panels added to baseline clinical model**

|  | **C-Index (95%CI)** | **NRI (95% CI)** | ***P* value** | **IDI (95% CI)** | ***P* value** | **LR-test,**  ***P* value** | **AIC** | **BIC** |
| --- | --- | --- | --- | --- | --- | --- | --- | --- |
| **AKI progression** |  |  |  |  |  |  |  |  |
| Base | 0.81(0.77-0.84) | Reference |  | Reference |  |  | 715 | 803 |
| Base+mFRI | 0.85 (0.82-0.89) | 0.24 (0.16-0.33) | <0.01 | 0.09 (0.06 - 0.11) | <0.001 | <0.001 | 646 | 740 |
| Base+mFRI+uNGAL/uCr | 0.87 (0.83-0.90) | 0.24 (0.14-0.34) | <0.01 | 0.13 (0.10 - 0.16) | <0.001 | <0.001 | 617 | 715 |
| Base+mFRI+uNGA/uCr | 0.87 (0.84-0.91) | 0.28 (0.19-0.38) | <0.01 | 0.14 (0.10 - 0.17) | <0.001 | <0.001 | 611 | 709 |
| **Progression to stage 3** |  |  |  |  |  |  |  |  |
| Base | 0.88(0.84-0.93) | Reference |  | Reference |  |  | 348 | 437 |
| Base+mFRI | 0.92(0.88-0.96) | 0.22 (0.10-0.34) | <0.01 | 0.08 (0.06 - 0.11) | <0.001 | <0.001 | 310 | 403 |
| Base+mFRI+uNGAL/Cr | 0.93(0.89-0.97) | 0.27 (0.13-0.41) | <0.01 | 0.16 (0.10 - 0.22) | <0.001 | <0.001 | 289 | 387 |
| Base+mFRI+uNGA/Cr | 0.93(0.90-0.97) | 0.23 (0.10-0.37) | <0.01 | 0.15 (0.09 - 0.21) | <0.001 | <0.001 | 289 | 387 |
| **Composite Outcome** |  |  |  |  |  |  |  |  |
| Base | 0.96(0.94-0.99) | Reference |  | Reference |  |  | 187 | 275 |
| Base+mFRI | 0.97(0.94-0.99) | 0.15 (0.03 - 0.28) | 0.01 | 0.06 (0.03 - 0.08) | <0.001 | <0.001 | 175 | 269 |
| Base+mFRI+uNGAL/uCr | 0.97(0.94-0.99) | 0.16 (-0.03-0.35) | 0.10 | 0.09 (0.03 - 0.14) | 0.001 | <0.001 | 173 | 272 |
| Base+mFRI+uNGA/uCr | 0.97(0.94-0.99) | 0.19 (0.03- 0.34) | 0.02 | 0.06 (0.04 - 0.09) | <0.001 | <0.001 | 176 | 274 |

The base clinical model was adjusted for age, gender, BMI, diabetes mellitus, hypertension, CAD, cerebrovascular disease, preoperative diuretic exposure, baseline eGFR, surgical type, cardiopulmonary bypass used, CVP, AKI stage at enrollment and SOFA score. AKI, acute kidney injury; AIC, Akaike information criterion, BIC, Bayesian information criterion; BMI, body mass index; CAD, coronary artery disease; CI, confidence interval; CVP, central venous pressure; eGFR, estimated glomerular filtration rate; IDI, integrated discrimination improvement; LR, Likelihood ratio; mFRI, modified furosemide responsiveness index; NRI, net reclassification improvement; OR, odds ratio; SOFA score, sequential organ failure assessment score, uCr, urinary creatinine; uNAG, urinary N-acetyl-β-D-glucosaminidase; uNGAL, urinary neutrophil gelatinase-associated lipocalin.
